# British Dietetic Association Guidelines for the Dietary Management of Chronic Constipation in Adults

**DOI:** 10.1111/jhn.70133

**Published:** 2025-10-13

**Authors:** Eirini Dimidi, Alice van der Schoot, Kevin Barrett, Adam D. Farmer, Miranda C. Lomer, S. Mark Scott, Kevin Whelan

**Affiliations:** ^1^ Department of Nutritional Sciences King's College London London UK; ^2^ New Road Surgery Rickmansworth UK; ^3^ Division of Gastroenterology & Hepatology St Louis University Hospital St Louis Missouri USA; ^4^ Department of Nutrition and Dietetics Guy's and St Thomas' NHS Foundation Trust London UK; ^5^ Wingate Institute of Neurogastroenterology, Blizard Institute Queen Mary University of London London UK

**Keywords:** constipation, diet, fibre, gut health, nutrition

## Abstract

**Background:**

Current clinical guidelines for chronic constipation offer limited dietary recommendations. The aim of this project was to develop the first comprehensive evidence‐based dietary guidelines for the management of chronic constipation in adults.

**Methods:**

Four systematic reviews and meta‐analyses were performed to identify eligible randomised controlled trials (RCTs). The findings generated from the meta‐analyses were then used to develop guideline statements using the Grading of Recommendations, Assessment, Development and Evaluation (GRADE) approach and a Delphi consensus survey among a multi‐disciplinary expert Guideline Steering Committee. Recommendation statements were produced for treatment response, stool output, gut symptoms, adverse events, and quality of life, and only based on the findings where ≥ 2 RCTs contributed to the meta‐analysis. The strength of recommendation was assessed using the GRADE approach. Consensus voting amongst the Guideline Steering Committee was performed using a modified Delphi survey approach.

**Results:**

The four systematic reviews included a total of 75 RCTs. Fifty‐nine dietary recommendation statements were generated and accepted through the Delphi survey. For dietary supplements, 15 recommendation statements relate to fibre supplements, 20 relate to probiotics, 2 to synbiotics, 5 to magnesium oxide, 2 to senna, and 3 to kiwifruit supplements. For foods, three recommendation statements related to kiwifruits, two to prunes, and two to rye bread. For drinks, five recommendation statements related to high mineral‐containing water. No recommendations were made for whole diet approaches due to a lack of evidence. Twelve statements had a very low level of evidence, 39 had a low level of evidence, and 8 had moderate evidence. Twenty‐seven statements were strong recommendations, and 32 were qualified recommendations.

**Conclusions:**

These are the first comprehensive evidence‐based dietary guidelines for the management of constipation based upon a robust systematic review and GRADE processes. Recommendations were made for dietary supplements, foods and drinks that have never been previously included in clinical guidelines, and can now be rapidly implemented into clinical practice, thereby improving clinical care and patient outcomes.

## Summary

- These are the first guidelines specifically for the dietary management of chronic constipation and are based on evidence from robust systematic reviews following the GRADE process.
- Psyllium supplements, certain probiotic strains, magnesium oxide supplements, kiwifruits, rye bread and high mineral water are recommended to improve specific constipation outcomes.
- No recommendations were made on whole diet approaches (e.g., high fibre diet) due to lack of evidence.

## 1 Introduction

Chronic constipation is characterised by unsatisfactory defecation that results from infrequent stools, difficult stool passage, or both [1]. It is a burdensome bowel disorder affecting 10.1% of the global population [2]. It significantly impacts quality of life and can result in considerable financial burden to both patients and healthcare systems [3, 4, 5]. Nearly all patients try lifestyle management options for symptom relief, including dietary modifications that primarily focus on increasing dietary fibre intake [6, 7]. However, more than half are not satisfied with their current treatment [6, 7], highlighting an urgent need to improve management strategies for chronic constipation.

Current therapeutic guidelines for chronic constipation recommend several dietary modifications for symptom relief [8, 9, 10]. However, there are considerable limitations with these guidelines, which may help explain the high treatment dissatisfaction rates [6, 7]. First, they focus on only a limited selection of dietary recommendations, primarily increasing fibre intake and adequate fluid intake, omitting other dietary strategies for which evidence on effectiveness exists. Second, some guidelines include dietary recommendations for which robust evidence either does not exist or does not support their use in constipation (e.g., fruits high in sorbitol) [10]. Third, the current guidelines are sometimes vague (e.g., ‘increase fibre intake’), lacking detailed information, for example, on specific evidence‐based foods that would help achieve the recommendations, and the optimal mode of administration. Therefore, there is a lack of comprehensive dietary guidelines for the management of chronic constipation that represent the totality of the available evidence and provide practical recommendations for clinical practice. This interferes with the advice given by healthcare professionals, ultimately negatively impacting patients' symptom management and overall satisfaction [6, 7].

To address this critical gap, we aimed to develop the first comprehensive evidence‐based dietary guidelines, focusing on all forms of dietary interventions (supplements, foods and drinks, whole diets), for the management of chronic constipation in adults, via a systematic review of the literature and a Delphi consensus process among an expert Guideline Steering Committee.

## 2 Materials and Methods

A Guideline Steering Committee consisting of seven experts in nutrition, dietetics, gut physiology and gastroenterology from primary, secondary and tertiary care was formed to determine the scope of the guidelines, including the target patient population, dietary interventions, critical outcomes, and the study design of eligible studies on which the guideline statements would be based. They also appraised the evidence based upon prospectively performed systematic reviews and meta‐analyses, contributed to Grading of Recommendations, Assessment, Development and Evaluation (GRADE) recommendation decisions, and participated in multiple rounds of the Delphi consensus process for the guideline statements.

### 2.1 Scope of the Dietary Guidelines

The guidelines are relevant to adults with chronic idiopathic constipation, who are otherwise considered healthy, and are aimed to cover evidence‐based recommendations for dietary supplements, foods and drinks, and whole diets, as and where evidence was available. Although studies that included solely people with secondary constipation were outside the scope of the guidelines, recommendations may also be considered for secondary constipation, but should be applied with caution, as this population was not assessed.

To comprehensively identify, review and appraise the existing evidence, four systematic reviews and meta‐analyses were undertaken [11, 12, 13, 14]. The findings from these meta‐analyses were then used to develop the guideline statements using the GRADE approach and a Delphi consensus survey among the Guideline Steering Committee.

### 2.2 Systematic Reviews and Meta‐Analyses for Evidence Generation

A Systematic Review Working Group consisting of three researchers from the Guideline Development Group (dietitians, nutritionists) was formed to undertake the systematic reviews. Four systematic reviews and meta‐analyses were undertaken to identify all relevant studies, quantify the effectiveness of identified dietary interventions in constipation outcomes, and critically appraise the evidence [11, 12, 13, 14]. These were conducted in line with the Cochrane Handbook for Systematic Reviews of Interventions and Preferred Reporting Items for Systematic reviews and Meta‐Analyses (PRISMA) updated guidelines [15, 16]. The eligibility criteria, search strategy, methods of screening, data extraction and analysis were determined in advance and detailed in protocols published in PROSPERO (CRD42020191404; CRD42021261670; CRD42021292029; CRD42021241072).

A Population, Intervention, Comparison and Outcomes framework was used (Table 1). In summary, participants with chronic idiopathic constipation, defined through either: (i) widely‐recognised clinical diagnostic criteria (e.g., the Rome criteria [17]); (ii) as defined by the author, clinician or participants (e.g., self‐reported constipation); or (iii) the presence of at least one symptom indicative of constipation (Table 1), were included. This broad definition of constipation ensures the guidelines are relevant to most people seeking treatment for their constipation‐related symptoms in primary, secondary or tertiary clinical practice. The following dietary interventions were explored and published as systematic reviews: fibre supplements [12], probiotic and synbiotic supplements [13], food, vitamin and mineral supplements [11], and foods, drinks and whole diets [14]. Only randomised controlled trials (RCTs) were included, where the dietary intervention was compared to a placebo control, to ensure the inclusion of robust trials [11, 12, 13]. The only exception was studies assessing foods, drinks and whole diets due to the challenges of placebos in this context [18] and because the evidence base was recognised to be less rigorous in this field, with very few RCTs with appropriate comparator groups. Relevant studies on foods, drinks and whole diets were eligible if they were an intervention trial (randomised, non‐randomised, controlled, non‐controlled) [14]. However, to ensure the guideline statements are based on robust evidence, statements were only generated for the evidence originating from RCTs.

**Table 1:** Table of inclusion and exclusion criteria for participants, intervention, comparator, outcomes and study designs.

Population	Intervention	Comparator	Outcomes	Study design
Adults (aged ≥ 18 years) with chronic idiopathic constipation identified through: (1) widely recognised clinical diagnostic criteria (e.g., Rome criteria); (2) as defined by the author, clinician or participants (e.g., self‐reported constipation); (3) presence of ≥ 1 of the following symptoms indicative of constipation: < 3 bowel movements per week, hard or lumpy stools, sensation of incomplete evacuation, straining, manual manoeuvres, physiological markers (e.g., slow gut transit time) or an evacuation disorder. Studies with inclusion criteria of ≥ 3 bowel movements per week and no other symptoms indicative of constipation were excluded. o restrictions on age, sex or ethnicity were applied. Community or outpatient settings were included. Studies were excluded if all participants had secondary constipation or belonged to specific clinical population groups (e.g., pregnant women, inpatients). However, a study was eligible if only a subset of the participants belonged to one of these groups.	**Fibre supplements:** Studies administering supplementary fibre defined by the Scientific Advisory Committee on Nutrition (2015). Individual or mixed fibre supplements, including prebiotic fibre, could be administered in pill, capsule, powder sachets, solutions or fortified food or drink forms (as long as the control group was such that the effect of the fibre alone could be isolated). Eligible dose was ≥ 3 g/d (Englyst method) or ≥ 4 g/d (AOAC method) for a minimum of 2 weeks. Studies of fibre supplements in conjunction with other interventions (e.g., dietary modification) were included only if the effect of the fibre alone could be isolated. Studies based on dietary advice to increase fibre intake as an intervention were excluded. **Probiotics:** Studies administering any species, strains and dose of live microorganisms, administered individually or as mixtures were eligible. **Synbiotics:** Studies administering any probiotic in conjunction with a prebiotic fibre administered together were eligible, as long as the dose of prebiotic fibre was > 1 g/d (Englyst method). Synbiotics based upon non‐prebiotic fibres (e.g., probiotics with polyphenols, probiotics with non‐prebiotic fibres) were excluded. Interventions could be administered in tablet, powder, capsule, softgel, fermented food or fortified food forms, as long as the control group was such that the effect of the probiotics or synbiotics alone could be isolated. Studies on probiotics or synbiotics in conjunction with other interventions (e.g., dietary modification) were included if the effect of the probiotics or synbiotics alone could be isolated. The minimum duration of intervention was 2 weeks, which was considered to be adequate to observe any potential benefits of the intervention on the outcomes of interest. **Food, vitamin or mineral supplements:** Studies administering food supplements as (i) an extract from a single food that is extracted using solvent‐extraction, water‐extraction, or other common extraction processes, or (ii) a freeze‐dried or other similarly prepared preparation of a single food; or individual vitamin or mineral supplements. Interventions were administered in supplement form, such as a tablet, powder, capsule or solution. Studies administering whole foods (e.g., fruits) were excluded. Studies administering supplements containing mixtures of foods, vitamins and/or mineral supplements (e.g., multivitamins) were excluded, as were supplements containing any other potentially active ingredients. Studies of food, vitamin or mineral supplements in conjunction with other interventions (e.g., laxatives, dietary modification) were included, as long as the effect of a food, vitamin or mineral supplement could be isolated. The minimum duration of intervention was 2 weeks. **Foods, drinks, herbs, spices and diets:** Studies administering individual foods, drinks, herbs, spices, or diets (defined as a change in habitual dietary pattern), that are widely available and applicable in practice were eligible. Studies were excluded if the intervention was a composite dish, due to difficulty in replicating multiple ingredients in a specific dosage in a clinical setting (e.g., a specific vegetable is eligible, but not a mixed vegetable soup), a combination of products that are not widely available in that form (e.g., prunes are eligible, but not a combination of yoghurt with prunes and linseeds), or a diet based on specially formulated composite foods (e.g., a Mediterranean diet is eligible, but not a diet based on a specially formulated soup).	An appropriate placebo control that allowed the effect of the intervention of interest alone to be isolated. Where the intervention was a fortified food or drink, an appropriate comparator was the same food or drink without the fibre/probiotic/synbiotic/food, vitamin or mineral supplement. Studies that contained multiple study arms were included if intervention and control arms could be isolated. For studies administering fibre supplements or synbiotics, the control interventions could contain a negligible amount of the fibre (< 0.5 g/d Englyst method or < 0.67 g/d AOAC method). Trials with or without a control arm were eligible, as follows: (i) no control arm; (ii) control arm with no intervention (e.g., no intervention or habitual diet); (iii) placebo control (e.g., placebo capsule, inert food, sham diet); (iv) comparator arm with a potentially active intervention (e.g., psyllium supplement, acupuncture, laxatives).	Studies reporting dichotomous or continuous data on response to treatment (treatment success), stool frequency (using bowel diary, stool collection, retrospective recall), stool consistency (using Bristol scale, other stool chart/diary or objective measure, stool collection, retrospective recall), physiological outcomes (e.g., stool weight, whole and regional gut transit time), frequency or severity of individual symptoms (e.g., straining, sense of completeness of evacuation, bloating; using symptom diary, visual analogues scales, Likert scale, etc.), Integrative symptom scores (using Patient Assessment of Constipation Symptoms, PAC‐SYM, etc.), symptom response (e.g., number of participants with three or more complete spontaneous bowel movements per week), quality of life (using Patient Assessment of Constipation Quality of Life, PAC‐QoL, etc.), laxative use, adverse events, compliance.	Randomised controlled trials with ≥ 2 study groups where it was possible to extract data on the intervention of interest and control. Parallel group and cross‐over studies with a washout period of ≥ 2 weeks were eligible. Cross‐over studies without an adequate washout period (< 2 weeks) were only considered eligible if the data from the first period could be extracted to reduce the risk of carryover effect. Intervention trials, including controlled and comparative trials (randomised or non‐randomised) or uncontrolled trials (e.g., single‐arm studies). Parallel group and crossover trials were eligible.

Searches of electronic databases took place between February 2022 and July 2023 (Table S1). The eligibility screening, data extraction and risk of bias assessment were conducted independently by two researchers, and any disparities at each stage were resolved through discussions with a third (E. D.) and fourth (K. W.) researcher. Meta‐analyses were performed where two or more RCTs of the same dietary intervention reported data for the same outcome (RevMan version 5.4, The Cochrane Collaboration, 2020). Dichotomous outcomes were expressed as risk ratio (RR) and 95% confidence interval (CI). Continuous outcomes were expressed as mean difference (MD) or standardised mean difference (SMD). Detailed descriptions of the methodology and findings of the four systematic reviews and meta‐analyses are reported elsewhere [11, 12, 13, 14].

### 2.3 Development of Recommendation Statements

Recommendation statements were produced for constipation‐related outcomes that were decided to be ‘critical’ by the Guideline Steering Committee (Table S2). Outcomes were converted into recommendation statements aggregated into five categories, as appropriate:

- Treatment response (number of people benefiting from the intervention);
- Stool output (stool frequency and consistency);
- Gut symptoms (global and individual symptoms);
- Adverse events (flatulence, bloating, abdominal pain/discomfort);
- Quality of life (global and specific components).

Recommendation statements were generated based solely on the findings of the four systematic reviews and meta‐analyses, where ≥ 2 RCTs contributed to the meta‐analysis [11, 12, 13, 14]. Statements were not generated for dietary interventions when only one study contributed to the meta‐analysis or for uncontrolled studies. This ensured the recommendation statements were based on as robust evidence as possible. Recommendation statements were developed for:

(a) overall dietary intervention types (i.e., ‘overall, fibre supplements…’, ‘overall, probiotics…’), when appropriate, and included all studies contributing to the meta‐analysis; and

(b) specific types of the dietary interventions (e.g., psyllium fibre, specific probiotic species or strains), where ≥ 2 RCTs contributed to the subgroup meta‐analysis.

### 2.4 GRADE

The GRADE was used to guide these recommendation statements, including the ‘level of evidence’ and the ‘strength of the recommendation’.

‘Level of evidence’ for each outcome (high, moderate, low, very low) was assessed using the GRADE approach. This represents the level of certainty/quality of evidence for a given outcome [19]. When several outcomes contributed to a statement (e.g., stool frequency and stool consistency for ‘stool output’ statement), the lowest rating among the individual outcomes was used for the overall level of evidence for that statement.

‘Strength of recommendation’ was also assessed using the GRADE approach. It represents the level of confidence that the desirable effects of a dietary intervention outweigh undesirable effects [19]. A ‘strong’ recommendation signifies that most or all people with chronic constipation will be best served by the recommended course of action. A ‘qualified’ recommendation signifies that not all people with chronic constipation will be best served by the recommended course of action, and there is a need to consider the individual′s circumstances, preferences and values more carefully than usual.

Each recommendation statement is assigned both a level of evidence and a strength of recommendation. Due to the different criteria used to determine these, it is possible that a statement may have *low level of evidence* due to, for example, inconsistency and indirectness of evidence, but have *a strong recommendation* due to, for example, applicability to most people with chronic constipation, benefits of use outweighing risks, moderate effect size, convenient use of dietary strategy and low cost.

Recommendations considered important to inform clinical practice (e.g., appropriate dosage), for which GRADE‐supported statements were not possible or applicable, were included as Good Practice Statements. These were either evidence‐based or based on the expert opinion of the Guideline Steering Committee.

### 2.5 Delphi Consensus Survey

Once initially generated, recommendation statements underwent consensus voting amongst the seven members of the Guideline Steering Committee using a multi‐stage online modified Delphi survey approach [20]. The Guideline Steering Committee had access to the full output data from the systematic reviews and meta‐analyses, both before and during the Delphi consensus voting. The committee members voted ‘agree’ or ‘disagree’ for each recommendation statement, with each member having equal voting rights, and members could also provide comments or suggestions. Only recommendation statements with ≥ 85% agreement among the Guideline Steering Committee were accepted. Any statements that did not reach 85% agreement were re‐formulated until ≥ 85% agreement was reached in consequent voting.

### 2.6 Guideline Endorsement

These dietary guidelines have been formally endorsed by the British Dietetic Association (BDA) through the society′s independent process. In addition, they have been approved by the UK Primary Care Society for Gastroenterology.

## 3 Results

**Figure 1:**
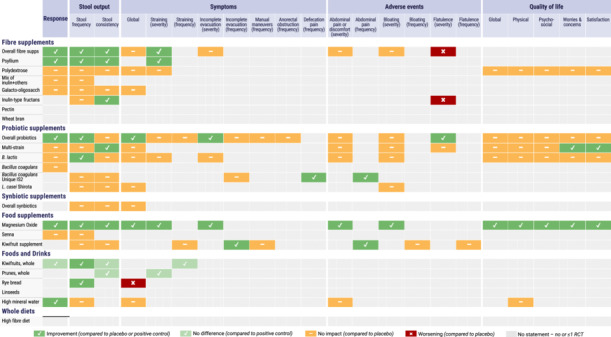
A clinician‐friendly summary guide of the recommendation statements as a practical tool that facilitates the adoption of the guidelines in clinical practice.

Overall, 59 recommendation statements were made relating to fibre supplements, probiotic supplements, synbiotic supplements, food supplements, and foods and drinks. No recommendations were made relating to whole diets due to a lack of evidence from the systematic reviews and meta‐analysis [14]. The recommendation statements are provided below, together with a brief summary of the supporting evidence, but for a more comprehensive summary, the original systematic reviews and meta‐analyses should be consulted. Table 2 provides the GRADE summary of findings. Figure 1 is a clinician‐friendly guide that summarises the evidence on a single page and can be used as a practical tool in clinical practice.

**Table 2:** Reasons for strength of recommendation for dietary strategies for the management of chronic constipation according to GRADE (Grading of Recommendations Assessment, Development and Evaluation) criteria.

Statement	Quality of evidence	Reason for quality of evidence grade	Recommendation	Patient groups	Benefits versus risks	Magnitude of effect	Values and preferences	Cost
*FIBRE SUPPLEMENTS*
1. Specific types of fibre supplements increase the number of people with constipation who have a clinical benefit.	Low	Downgraded due to high risk of bias, inconsistency, and imprecision. Upgraded for dose‐response effect.	Strong	Applicable to most people with chronic constipation. Studies diagnosed constipation using the Rome II, III and IV criteria, clinician‐defined criteria, slow transit constipation or presence of specific symptoms (infrequent bowel movements and/or hard stools). One study also included IBS‐C (along with chronic constipation), diagnosed using presence of specific symptoms. Outcome was assessed as proportion of people with constipation relief; improvement in global constipation symptoms; no straining; increase of ≥ 1 bowel movement/week.	Fibre may increase severity of flatulence. Adverse events, including other gastrointestinal symptoms, have been reported across studies.	Moderate (RR: 1.48, 95% CI: 1.17, 1.88).	Easy and convenient. Some patients may not like using supplements.	Inexpensive
1.1. Psyllium supplements increase the number of people with constipation who have a clinical benefit.	Low	Downgraded due to high risk of bias, imprecision.	Strong	Applicable to most people with chronic constipation. Studies diagnosed constipation using the Rome IV criteria, clinician‐defined criteria, or specific symptoms (infrequent bowel movements and/or hard stools). One study also included IBS‐C (along with chronic constipation), diagnosed using presence of specific symptoms. Outcome was assessed as proportion of people with improvement in global constipation symptoms or no straining.	Adverse events of gastrointestinal symptoms have been reported in one study.	Moderate (RR: 1.82, 95% CI: 1.51, 2.20).		
1.2. Polydextrose, mixtures of inulin and other fibres, and galacto‐oligosaccharide supplements do not impact the number of people with constipation who have a clinical benefit.	Low	Polydextrose: Downgraded due to imprecision. Mixtures of inulin and other fibres: Downgraded due to high risk of bias, inconsistency, and imprecision. Galacto‐oligosaccharides: Downgraded due to imprecision.	Strong	Applicable to most people with chronic constipation. Polydextrose: Studies diagnosed constipation using the Rome IV criteria. Outcome was assessed as proportion of people with constipation relief. Mixtures of inulin and other fibres: Studies diagnosed constipation using Rome II criteria or presence of specific symptoms (infrequent bowel movements). Outcome was assessed as proportion of people with no straining or constipation relief. Galacto‐oligosaccharides: Studies diagnosed constipation using the Rome IV criteria Outcome was assessed as proportion of people with increase of ≥ 1 bowel movement/week.	Polydextrose: Adverse events of gastrointestinal symptoms have been reported. Mixtures of inulin and other fibres: may increase severity of flatulence. Galacto‐oligosaccharides: well tolerated, but may lead to bloating/flatulence due to prebiotic action.	Polydextrose: No effect (RR: 1.07, 95% 0.83, 1.39). Mixtures of inulin and other fibres: No effect (RR: 1.64, 95% 0.61, 4.42). Galacto‐oligosaccharides: No effect (RR: 1.51, 95% CI: 0.94, 2.45).		
2. Specific types of fibre supplements increase stool frequency and soften stool consistency in constipation.	Low	Stool frequency: Downgraded due to high risk of bias and inconsistency. Upgrade due to dose response effect. Stool consistency: Downgraded due to high risk of bias, imprecision, possible publication bias. Upgraded due to dose–response effect.	Strong	Applicable to most people with chronic constipation. Stool frequency: Studies diagnosed constipation using the Rome II, III and IV criteria and other clinical criteria (CCCS score), clinician‐defined criteria, slow transit constipation, or presence of specific symptoms (infrequent bowel movements, hard stools, straining and/or feeling of incomplete evacuation). One study also included IBS‐C (along with chronic constipation), diagnosed using presence of specific symptoms. Stool consistency: Studies diagnosed constipation using the Rome II, III and IV criteria and other clinical criteria (CCCS score), clinician‐defined criteria, or presence of specific symptoms (infrequent bowel movements, hard stools, and/or straining). One study also included IBS‐C (along with chronic constipation), diagnosed using presence of specific symptoms.	Fibre may increase severity of flatulence. Adverse events, including other gastrointestinal symptoms, have been reported across studies.	Stool frequency: Moderate (SMD: 0.72, 95% CI: 0.36, 1.08). Stool consistency: Small (SMD: 0.32, 95% CI: 0.18, 0.46).		
2.1. Psyllium supplements increase stool frequency and soften stool consistency in constipation.	Low	Stool frequency: Downgraded due to high risk of bias and imprecision. Upgraded due to large magnitude of effect. Stool consistency: Downgraded due to high risk of bias and imprecision.	Strong	Applicable to most people with chronic constipation. Stool frequency and consistency: Studies diagnosed constipation using clinician‐defined criteria or presence of specific symptoms (infrequent bowel movements, hard stools). One study also included IBS‐C (along with chronic constipation), diagnosed using presence of specific symptoms.	Adverse events of gastrointestinal symptoms have been reported in one study.	Stool frequency: large (SMD: 1.13, 95% CI: 0.39, 1.88). Stool consistency: moderate (SMD: 0.52, 95% CI: 0.25, 0.78).		
2.2. Inulin‐type fructan supplements do not impact stool frequency in constipation, but soften stool consistency.	Low	Stool frequency and inconsistency: Downgraded due to high risk of bias and imprecision.	Qualified	Applicable to most people with chronic constipation. Stool frequency and consistency: Studies diagnosed constipation using presence of specific symptoms (infrequent bowel movements, hard stools, and/or feeling of incomplete evacuation).	Inulin‐type fructans may increase severity of flatulence.	Stool frequency: no effect (SMD: 0.76, 96% CI: −0.19, 1.71). Stool consistency: Small (SMD: 0.36, 95% CI: 0.03, 0.70).		
2.3. Mixtures of inulin and other fibre supplements do not impact stool frequency in constipation.	Very low	Downgraded due to high risk of bias, inconsistency, and imprecision.	Strong	Applicable to most people with chronic constipation. Studies diagnosed constipation using Rome II criteria or presence of specific symptoms (infrequent bowel movements).	May increase severity of flatulence.	No effect (SMD: 0.37, 95% CI: −0.85, 1.60).		
2.4. Polydextrose and galacto‐oligosaccharide supplements do not impact stool frequency or stool consistency in constipation.	Low	Polydextrose: Stool frequency and consistency: downgraded due to imprecision. Galacto‐oligosaccharides: Stool frequency and consistency: downgraded due to inconsistency and imprecision.	Strong	Applicable to most people with chronic constipation. Polydextrose: Stool frequency and consistency: studies diagnosed constipation using the Rome III criteria or clinician‐defined criteria. Galacto‐oligosaccharides: Stool frequency and consistency: studies diagnosed constipation using the Rome IV criteria or presence of specific symptoms (infrequent bowel movements, hard stools).	Polydextrose: Adverse events of gastrointestinal symptoms have been reported. Galacto‐oligosaccharides: well tolerated, but may lead to bloating/flatulence due to prebiotic action.	Polydextrose: Stool frequency: no effect (SMD: −0.03, 95% CI: −0.28, 0.23). Stool consistency: no effect (SMD: 0.19, 95% CI: −0.06, 0.45). Galacto‐oligosaccharides: Stool frequency: no effect (SMD: 0.62, 95% CI: −0.10, 1.34). Stool consistency: no effect (SMD: 0.16, 95% CI: −0.34, 0.66).		
3. Overall, fibre supplements do not impact global symptoms of constipation, but specific types of fibre supplements improve specific symptoms of constipation.	Low	Global symptoms: Downgraded due to high risk of bias. Straining (severity): Downgraded due to high risk of bias and imprecision. Upgraded for dose response effect. Incomplete evacuation (severity): Downgraded due to high risk of bias and imprecision.	Qualified	Applicable to most people with chronic constipation. Global symptoms: Studies diagnosed constipation using the Rome III and IV criteria, other clinical diagnostic criteria (CCCS), and slow transit constipation. Straining (severity): Studies diagnosed constipation using the Rome III criteria, clinician‐defined criteria, or presence of specific symptoms (infrequent bowel movements). Incomplete evacuation (severity): Studies diagnosed constipation using the presence of specific symptoms (infrequent bowel movements).	Fibre may increase severity of flatulence. Adverse events, including other gastrointestinal symptoms, have been reported across studies.	Global symptoms: no effect (SMD: −0.15, 95% CI: −0.39, 0.08). Straining (severity): small (SMD: −0.32, 95% CI: −0.59, −0.05). Incomplete evacuation (severity): no effect (SMD: −0.02, 95% CI: −0.39, 0.35).		
3.1. Psyllium supplements reduce the severity of straining in constipation.	Low	Downgraded due to high risk of bias and imprecision.	Strong	Applicable to most people with chronic constipation. Studies diagnosed constipation using clinician‐defined criteria, or presence of specific symptoms (infrequent bowel movements).	Adverse events of gastrointestinal symptoms have been reported in one study.	Moderate (SMD: −0.65, 95% CI: −0.91, −0.39).		
3.2. Polydextrose supplements do not impact global symptoms or specific symptoms of constipation.	Low	Global symptoms and straining (severity): Downgraded due to imprecision.	Strong	Applicable to most people with chronic constipation. Global symptoms: Studies diagnosed constipation using the Rome III criteria or clinician‐defined criteria (CCCS). Straining (severity): Studies diagnosed constipation using the Rome III criteria.	Polydextrose: Adverse events of gastrointestinal symptoms have been reported.	Global symptoms: no effect (SMD: −0.09, 95% CI: −0.34, 0.17). Straining (severity): no effect (SMD: −0.05, 95% CI: −0.38, 0.27).		
3.3. Galacto‐oligosaccharide supplements do not impact global symptoms of constipation.	Low	Downgraded due to imprecision.	Strong	Applicable to most people with chronic constipation. Studies diagnosed constipation using the Rome IV criteria.	Well tolerated, but may lead to bloating/flatulence due to prebiotic action.	No effect (SMD: −0.04, 95% CI: −0.41, 0.32).		
4. Specific types of fibre supplements increase the severity of flatulence in constipation, but not bloating or abdominal pain/discomfort.	Low	Flatulence, bloating, abdominal pain/discomfort: Downgraded due to high risk of bias and imprecision.	Strong	Applicable to most people with chronic constipation. Flatulence: Studies diagnosed constipation using a slow transit constipation diagnosis or the presence of specific symptoms (infrequent bowel movements). Bloating: Studies diagnosed constipation using the Rome III criteria and/or slow transit constipation, or the presence of specific symptoms (infrequent bowel movements). Abdominal pain/discomfort: Studies diagnosed constipation using clinician‐defined criteria, slow transit constipation diagnosis or the presence of specific symptoms (infrequent bowel movements).	Fibre may increase severity of flatulence. Adverse events, including other gastrointestinal symptoms, have been reported across studies.	Flatulence: large (SMD: 0.80, 95% CI: 0.47, 1.13). Bloating: no effect (SMD: 0.07, 95% CI: −0.38, 0.51). Abdominal pain/discomfort: no effect (SMD: −0.14, 95% CI: −0.36, 0.09).		
4.1. Inulin‐type fructan supplements increase the severity of flatulence in constipation, but not bloating.	Low	Flatulence and bloating: Downgraded due to high risk of bias and imprecision.	Strong	Applicable to most people with chronic constipation. Studies diagnosed constipation using the presence of specific symptoms (infrequent bowel movements).	Inulin‐type fructans may increase severity of flatulence.	Flatulence: large (SMD: 0.79, 95% CI: 0.44, 1.14). Bloating: no effect (SMD: 0.25, 95% CI: −0.08, 0.59).		
5. Polydextrose supplements do not impact global or specific components of quality of life in constipation.	Moderate	All downgraded for imprecision.	Strong	Applicable to most people with chronic constipation. Studies diagnosed constipation using the Rome IV criteria and other clinical diagnostic criteria (CCCS).	Polydextrose: Adverse events of gastrointestinal symptoms have been reported.	Global: no effect (MD: −0.04, 95% CI: −0.19, 0.10). Satisfaction: no effect (MD: −0.05, 95% CI: −0.32, 0.23). Physical discomfort: no effect (MD: 0.12, 95% CI: −0.08, 0.32). Worries and concerns: no effect (MD: −0.04, 95% CI: −0.21, 0.12). Psychosocial discomfort: no effect (MD: −0.04, 05% CI: −0.18, 0.11).		
*PROBIOTIC SUPPLEMENTS*
6. Probiotics overall may increase the number of people with constipation who have a clinical benefit, though it is unclear which species or strains are effective.	Low	Downgraded due to high risk of bias and imprecision.	Qualified	Applicable to most people with chronic constipation. Studies diagnosed constipation using the Rome III and IV criteria, other clinical criteria (CCCS), and/or presence of specific symptoms (infrequent bowel movements and/or hard stools). One study also included IBS‐C (along with chronic constipation), diagnosed using the Rome III criteria. One study did not report method of diagnosis. Outcome was assessed as proportion of people with normal stool consistency; patient‐reported treatment success; satisfactory relief of constipation; > 25% reduction in gut transit time; ≥ or > 3 bowel movements/week and/or increase of ≥ 1 bowel movement/week; improvement in constipation‐related quality of life; improvement in global constipation symptoms; no longer meeting the Rome III criteria.	Adverse events, including gastrointestinal symptoms, were reported across studies in both the probiotic and control groups. However, the number of events was similar between groups, and adverse events were reported in few studies only.	Small (RR: 1.28, 95% CI: 1.07, 152).	Most probiotics are easy and convenient to take. Some patients may not like using supplements. Effects are strain‐specific, and it may be challenging to find probiotic strain with evidence in the local market.	May be expensive
6.1. Multi‐strain probiotic supplements, *Bifidobacterium lactis* supplements, and *Bacillus coagulans* supplements do not impact the number of people with constipation who have a clinical benefit.	Low	Multi‐strain probiotics and *B. lactis*: Downgraded due to high risk of bias and imprecision. *B. coagulans*: Downgraded for imprecision.	Qualified	Applicable to most people with chronic constipation. Multi‐strain probiotics: Studies diagnosed constipation using the Rome III and IV criteria. One study also included IBS‐C (along with chronic constipation), diagnosed using the Rome III criteria. Outcome was assessed as proportion of people with normal stool consistency; > 3 bowel movements/weeks; improvement in constipation‐related quality of life. *B. lactis*: Studies diagnosed constipation using the Rome III criteria, and/or presence of specific symptoms (infrequent bowel movements and/or hard stools). Outcome was assessed as proportion of people with satisfactory relief of constipation; > 3 bowel movements/weeks; improvement in global constipation symptoms; no longer meeting the Rome III criteria. *B. coagulans*: Studies diagnosed constipation using the Rome III criteria. Outcome was assessed as proportion of people with normal stool consistency; > 25% reduction in gut transit time.	Adverse events, including gastrointestinal symptoms, were reported across studies in both the probiotic and control groups. However, the number of events was similar between groups, and adverse events were reported in few studies only.	Multi‐strain probiotics: No effect (RR: 1.02, 95% CI: 0.74, 1.41). *B. lactis*: No effect (RR: 1.14, 95% CI: 0.81, 1.60). *B. coagulans*: No effect (RR: 1.57, 95% CI: 0.93, 2.67).		
7. Probiotics overall, and some specific species, increase stool frequency, but do not impact stool consistency in constipation.	Low	Stool frequency: Downgraded due to high risk of bias and inconsistency. Stool consistency: Downgraded due to high risk of bias and potential publication bias.	Qualified	Applicable to most people with chronic constipation. Stool frequency: Studies diagnosed constipation using the Rome II, III and IV criteria, other clinical criteria (Chinese Constipation questionnaire), presence of evacuation disorder, and/or presence of specific symptoms (infrequent bowel movements and/or hard stools). Two studies also included IBS‐C (along with chronic constipation), diagnosed using the Rome III or IV criteria. Two studies did not report method of diagnosis. Stool consistency: Studies diagnosed constipation using the Rome II, III and IV criteria, other clinical criteria (CCCS, Chinese Constipation questionnaire), presence of evacuation disorder, and/or presence of specific symptoms (infrequent bowel movements and/or hard stools). One study also included IBS‐C (along with chronic constipation), diagnosed using the Rome III or IV criteria. One study did not report method of diagnosis.	Adverse events, including gastrointestinal symptoms, were reported across studies in both the probiotic and control groups. However, the number of events was similar between groups, and adverse events were reported in few studies only.	Stool frequency: Moderate (SMD: 0.71, 95% CI: 0.37, 1.04). Stool consistency: No effect (SMD: 0.26, 95% CI: −0.03, 0.54).		
7.1. Multi‐strain probiotic supplements do not impact stool frequency in constipation, but soften stool consistency.	Low	Stool frequency: Downgraded due to high risk of bias. Stool consistency: Downgraded due to high risk of bias and imprecision.	Qualified	Applicable to most people with chronic constipation. Stool frequency: Studies diagnosed constipation using the Rome III or IV criteria, presence of evacuation disorder, and/or presence of specific symptoms (hard stools). Two studies also included IBS‐C (along with chronic constipation), diagnosed using the Rome III or IV criteria. Stool consistency: Studies diagnosed constipation using the Rome III or IV criteria, presence of evacuation disorder, and/or presence of specific symptoms (hard stools). One study also included IBS‐C (along with chronic constipation), diagnosed using the Rome IV criteria.	Adverse events, including gastrointestinal and skin symptoms, were reported in both the multi‐strain probiotic and control groups in one study. However, the number of events was similar between groups.	Stool frequency: No effect (SMD: 0.30, 95% CI: −0.07, 0.67). Stool consistency: Small (SMD: 0.27, 95% CI: 0.03, 0.51).		
7.2. *Bifidobacterium lactis* supplements increase stool frequency in constipation, but do not impact stool consistency.	Moderate	Stool frequency and consistency: Downgraded due to high risk of bias.	Qualified	Applicable to most people with chronic constipation. Stool frequency: Studies diagnosed constipation using the Rome III criteria, presence of evacuation disorder, and/or presence of specific symptoms (infrequent bowel movements and/or hard stools). One study did not report method of diagnosis. Stool consistency: Studies diagnosed constipation using the Rome III criteria, presence of evacuation disorder, and/or presence of specific symptoms (infrequent bowel movements and/or hard stools).	Adverse events, including gastrointestinal symptoms, were reported in one study in both the *B. lactis* and control groups. However, the number of events was similar between groups.	Stool frequency: moderate (SMD: 0.48, 95% CI: 0.19, 0.77). Stool consistency: no effect (SMD: 0.25, 95% CI: −0.08, 0.59).		
7.3. *Bacillus coagulans* Unique IS2 supplements do not impact stool frequency or stool consistency in constipation.	Low	Stool frequency and consistency: Downgraded due to inconsistency and imprecision.	Qualified	Applicable to most people with chronic constipation. Stool frequency and consistency: Studies diagnosed constipation using the Rome III.	Well tolerated	Stool frequency: no effect (SMD: 1.24, 95% CI: −0.37, 2.84). Stool consistency: no effect (SMD: 0.07, −1.06, 1.19).		
7.4. *Lactobacillus casei* Shirota supplements do not impact stool frequency or stool consistency in constipation.	Very low	Stool frequency and consistency: Downgraded due to high risk of bias, inconsistency and imprecision.	Strong	Applicable to most people with chronic constipation. Stool frequency and consistency: Studies diagnosed constipation using the Rome II criteria, other clinical criteria (Chinese Constipation questionnaire), and/or presence of specific symptoms (infrequent bowel movements and/or hard stools). One study did not report method of diagnosis.	Adverse events, including gastrointestinal symptoms, were reported in one study in both the *L. casei* Shirota and control groups. However, the number of events was similar between groups.	Stool frequency: no effect (SMD: 0.61, 95% CI: −0.53, 1.74). Stool consistency: no effect (SMD: 1.10, 95% CI: −0.39, 2.59).		
8. Probiotics overall improve global symptoms of constipation, but not the majority of individual symptoms, though it is unclear which species or strains are effective.	Very low	Global: Downgraded due to high risk of bias and inconsistency. Straining (severity): Downgraded due to high risk of bias. Incomplete evacuation (severity): Downgraded due to high risk of bias. Upgraded due to large magnitude of effect. Straining, manual manoeuvres, anorectal obstruction (frequency): Downgraded due to high risk of bias and imprecision. Incomplete evacuation (frequency): Downgraded due to high risk of bias, inconsistency and imprecision. Manual manoeuvres (frequency): Downgraded due to high risk of bias and imprecision.	Qualified	Applicable to most people with chronic constipation. Global:Studies diagnosed constipation using the Rome II, III and IV criteria, other clinical criteria (CCCS, Chinese Constipation questionnaire), and/or presence of specific symptoms (infrequent bowel movements and/or hard stools). Two studies also included IBS‐C (along with chronic constipation), diagnosed using the Rome III or IV criteria. One study did not report method of diagnosis. Straining (severity; frequency): Studies diagnosed constipation using the Rome III criteria. Incomplete evacuation (severity): Studies diagnosed constipation using the Rome II or III criteria, other clinical criteria (CCCS, Chinese Constipation questionnaire), presence of evacuation disorder, and/or presence of specific symptoms (hard stools). Incomplete evacuation, manual manoeuvres, anorectal obstruction (frequency): Studies diagnosed constipation using the Rome III criteria, and/or presence of specific symptoms (hard stools).	Adverse events, including gastrointestinal symptoms, were reported across studies in both the probiotic and control groups. However, the number of events was similar between groups, and adverse events were reported in few studies only.	Global: moderate (SMD: −0.46, 95% CI: −0.89, −0.04). Straining (severity): no effect (SMD: −0.11, 05% CI: −0.32, 0.10). Straining (frequency): no effect (SMD: −0.48, 95% CI: −1.48, 0.52). Incomplete evacuation (severity): large (SMD: −0.81, 95% CI: −1.17, −0.45). Incomplete evacuation (frequency): no effect (SMD: −0.41, 95% CI: −1.18, 0.36). Manual manoeuvres (frequency): no effect (SMD: −0.26, 95% CI: −0.82, 0.30). Anorectal obstruction (frequency): no effect (SMD: −0.31, −1.13, 0.51).		
8.1. Multi‐strain probiotic supplements do not impact global symptoms of constipation.	Low	Downgraded due to high risk of bias and imprecision.	Qualified	Applicable to most people with chronic constipation. Studies diagnosed constipation using the Rome III and IV criteria. Two studies also included IBS‐C (along with chronic constipation), diagnosed using the Rome III or IV criteria.	Adverse events, including gastrointestinal and skin symptoms, were reported in both the multi‐strain probiotic and control groups in one study. However, the number of events was similar between groups.	No effect (SMD: −0.04, 95% CI: −0.53, 0.44).		
8.2. *Bifidobacterium lactis* supplements do not impact global symptoms or specific symptoms of constipation.	Low	Global: Downgraded due to imprecision. Straining (severity): Downgraded due to imprecision. Incomplete evacuation (severity): Downgraded due to high risk of bias and imprecision.	Qualified	Applicable to most people with chronic constipation. Global: Studies diagnosed constipation using the Rome III criteria, and/or presence of specific symptoms (infrequent bowel movements and/or hard stools). Straining (severity): Studies diagnosed constipation using the Rome III criteria. Incomplete evacuation (severity): Studies diagnosed constipation using the Rome III criteria, presence of evacuation disorder, and/or presence of specific symptoms (hard stools).	Adverse events, including gastrointestinal symptoms, were reported in one study in both the *B. lactis* and control groups. However, the number of events was similar between groups.	Global: no effect (SMD: 0.08, 95% CI: −0.16, 0.32). Straining (severity): no effect (SMD: −0.16, 95% CI: −0.41, 0.09). Incomplete evacuation (severity): no effect (SMD: −0.42, 95% CI: −1.07, 0.22).		
8.3. *B. coagulans* Unique IS2 supplements improve specific symptoms of constipation.	Low	Incomplete evacuation, defecation pain (frequency): Downgraded due to inconsistency and imprecision.	Qualified	Applicable to most people with chronic constipation. Incomplete evacuation (frequency); defecation pain (frequency): Studies diagnosed constipation using the Rome III criteria.	Well tolerated.	Incomplete evacuation (frequency): no effect (SMD: −0.67, 95% CI: −1.40, 0.10). Defecation pain (frequency): small (MD: −0.64 points in 5‐point Likert scale, 95% CI: −1.27, −0.02).		
8.4. *Lactobacillus casei* Shirota supplements do not impact global symptoms of constipation.	Very low	Downgraded due to high risk of bias, inconsistency, and imprecision.	Strong	Applicable to most people with chronic constipation. Studies diagnosed constipation using the Rome II criteria, or other clinical criteria (Chinese Constipation questionnaire). One study did not report method of diagnosis.	Adverse events, including gastrointestinal symptoms, were reported in one study in both the *L. casei* Shirota and control groups. However, the number of events was similar between groups.	No effect (SMD: −1.17, 95% CI: −2.73, 0.39).		
9. Probiotics overall reduce the severity of flatulence in constipation, but do not impact abdominal pain/discomfort or bloating, though it is unclear which species or strains are effective.	Low	Flatulence: Downgraded due to high risk of bias and imprecision. Abdominal pain/discomfort: Downgraded due to high risk of bias. Bloating: Downgraded due to high risk of bias and inconsistency.	Qualified	Applicable to most people with chronic constipation. Flatulence: Studies diagnosed constipation using the Rome III criteria, and/or presence of specific symptoms (hard stools). One study also included IBS‐C (along with chronic constipation), diagnosed using the Rome III criteria. Abdominal pain/discomfort: Studies diagnosed constipation using the Rome III criteria. One study also included IBS‐C (along with chronic constipation), diagnosed using the Rome III criteria. Bloating: Studies diagnosed constipation using the Rome II and III criteria, other clinical criteria (Chinese Constipation questionnaire), presence of evacuation disorder, and/or presence of specific symptoms (hard stools). One study also included IBS‐C (along with chronic constipation), diagnosed using the Rome III criteria. One study did not report method of diagnosis.	Adverse events, including gastrointestinal symptoms, were reported across studies in both the probiotic and control groups. However, the number of events was similar between groups, and adverse events were reported in few studies only.	Flatulence: small (SMD: −0.37, 95% CI: −0.73, −0.00). Abdominal pain/discomfort: no effect (SMD: −0.10, 95% CI: −0.28, 0.09). Bloating: no effect (SMD: −0.38, 95% CI: −0.82, 0.06).		
9.1. Multi‐strain probiotic supplements do not impact the severity of flatulence, abdominal pain/discomfort or bloating in constipation.	Very low	Flatulence: Downgraded due to high risk of bias and imprecision. Abdominal pain/discomfort: Downgraded due to high risk of bias and imprecision. Bloating: Downgraded due to high risk of bias, inconsistency, and imprecision.	Qualified	Applicable to most people with chronic constipation. Flatulence; abdominal pain/discomfort: Studies diagnosed constipation using the Rome III and IV criteria. One study also included IBS‐C (along with chronic constipation), diagnosed using the Rome III criteria. Bloating: Studies diagnosed constipation using the Rome III, presence of evacuation disorder, and/or presence of specific symptoms (hard stools). One study also included IBS‐C (along with chronic constipation), diagnosed using the Rome III criteria.	Adverse events, including gastrointestinal and skin symptoms, were reported in both the multi‐strain probiotic and control groups in one study. However, the number of events was similar between groups.	Flatulence: no effect (SMD: −0.24, 95% CI: −0.62, 0.14). Abdominal pain/discomfort: no effect (SMD: −0.19, 95% CI: −0.56, 0.18). Bloating: no effect (SMD: −0.63, 95% CI: −1.76, 0.51).		
9.2. *Bifidobacterium lactis* supplements do not impact the severity of abdominal pain or bloating in constipation.	Very low	Abdominal pain: Downgraded due to imprecision. Bloating: Downgraded due to high risk of bias, inconsistency, and imprecision.	Qualified	Applicable to most people with chronic constipation. Abdominal pain: Studies diagnosed constipation using the Rome III criteria. Bloating: Studies diagnosed constipation using the Rome III criteria, presence of evacuation disorder, and/or presence of specific symptoms (hard stools).	Adverse events, including gastrointestinal symptoms, were reported in one study in both the *B. lactis* and control groups. However, the number of events was similar between groups.	Abdominal pain: no effect (SMD: −0.10, 95% CI: −0.28, 0.09). Bloating: no effect (SMD: −0.39, 95% CI: −1.25, 0.46).		
9.3. *Bacillus coagulans* Unique IS‐2 supplements decrease frequency of abdominal pain in constipation.	Low	Downgraded due to inconsistency and imprecision.	Qualified	Applicable to most people with chronic constipation. Studies diagnosed constipation using the Rome III criteria.	Well tolerated.	Small (MD: −0.67 points in a 5‐point Likert scale, 95% CI: −1.14, −0.20).		
9.4. *Lactobacillus casei* Shirota supplements do not impact the severity of bloating in constipation.	Low	Downgraded due to high risk of bias and imprecision.	Strong	Applicable to most people with chronic constipation. Studies diagnosed constipation using the Rome II criteria, or other clinical criteria (Chinese Constipation questionnaire). One study did not report method of diagnosis.	Adverse events, including gastrointestinal symptoms, were reported in one study in both the *L. casei* Shirota and control groups. However, the number of events was similar between groups.	No effect (SMD: −0.12, 95% CI: −0.42, 0.18).		
10. Probiotics overall do not impact global or specific components of quality of life in constipation.	Low	All downgraded due to high risk of bias and imprecision.	Qualified	Applicable to most people with chronic constipation. Studies diagnosed constipation using the Rome III criteria, and/or presence of specific symptoms (infrequent bowel movements and/or hard stools). One study also included IBS‐C (along with chronic constipation), diagnosed using the Rome III criteria.	Adverse events, including gastrointestinal symptoms, were reported across studies in both the probiotic and control groups. However, the number of events was similar between groups, and adverse events were reported in few studies only.	Global: no effect (MD: −0.13 points on PAC‐QoL, 95% CI: −0.36, 0.10). Satisfaction: no effect (MD: 0.52 points on PAC‐QoL, 95% CI: −0.35, 1.38). Physical discomfort: no effect (MD: −0.06 points on PAC‐QoL, 95% CI: −0.30, 0.18). Psychosocial: no effect (MD: −0.05 points on PAC‐QoL, 95% CI: −0.17, 0.07). Worries and concerns: no effect (MD: −0.37 points on PAC‐QoL, 95% CI: −0.91, 0.17).		
10.1. Multi‐strain probiotic supplements do not impact global quality of life in constipation, but improve specific components of quality of life.	Low	All downgraded due to high risk of bias and imprecision.	Qualified	Applicable to most people with chronic constipation. Studies diagnosed constipation using the Rome III criteria, and/or presence of specific symptoms (hard stools). One study also included IBS‐C (along with chronic constipation), diagnosed using the Rome III criteria.	Adverse events, including gastrointestinal and skin symptoms, were reported in both the multi‐strain probiotic and control groups in one study. However, the number of events was similar between groups.	Global: no effect (MD: −0.25 points on PAC‐QoL, 95% CI: −1.05, 0.54). Satisfaction: moderate (MD: 1.62 points on PAC‐QoL, 95% CI: 1.36, 1.88). Physical discomfort: no effect (MD: −0.23 points on PAC‐QoL, 95% CI: −1.12, 0.66). Psychosocial: no effect (MD: −0.09 points on PAC‐QoL, 95% CI: −0.36, 0.17). Worries and concerns: small (MD: −1.19 points PAC‐QoL, 95% CI: −1.44, −0.94).		
10.2. *Bifidobacterium lactis *supplements do not impact global or specific components of quality of life in constipation.	Moderate	All downgraded due to imprecision.	Qualified	Applicable to most people with chronic constipation. Studies diagnosed constipation using the Rome III criteria, and/or presence of specific symptoms (infrequent bowel movements, hard stools).	Adverse events, including gastrointestinal symptoms, were reported in one study in both the *B. lactis* and control groups. However, the number of events was similar between groups.	Global: no effect (MD: −0.03 points on PAC‐QoL, 95% CI: −0.14, 0.08). Satisfaction: no effect (MD: 0.02 points on PAC‐QoL, 95% CI: −0.19, 0.22). Physical discomfort: no effect (MD: 0.05, points on PAC‐QoL, 95% CI: −0.15, 0.24). Psychosocial: no effect (MD: −0.04 points on PAC‐QoL, 95% CI: −0.17, 0.09). Worries and concerns: no effect (MD: −0.11 points on PAC‐QoL, 95% CI: −0.27, 0.04).		
*SYNBIOTIC SUPPLEMENTS*
11. Synbiotics do not impact stool frequency or stool consistency in constipation.	Low	Downgraded due to inconsistency and imprecision.	Qualified	Applicable to most people with chronic constipation. Stool frequency and consistency: Studies diagnosed constipation using the Rome III criteria.	Well tolerated, but may lead to bloating/flatulence due to prebiotic action.	Stool frequency: no effect (MD: 0.54 bowel movements/week, 95% CI: −0.80, 1.87). Stool consistency: no effect (SMD: 0.16, 95% CI: −0.48, 0.81).	Easy and convenient to take. Some patients may not like using supplements.	May be expensive
12. Synbiotics do not impact global symptoms of constipation.	Very low	Downgraded due to high risk of bias, inconsistency, and imprecision.	Qualified	Applicable to most people with chronic constipation. Studies diagnosed constipation using the Rome III criteria.		No effect (SMD: −0.55, 95% CI: −1.57, 0.48).		
*FOOD SUPPLEMENTS*								
*Magnesium oxide supplements*								
13. Magnesium oxide supplements increase the number of people with constipation who have a clinical benefit.	Moderate	Downgraded due to imprecision.	Strong	Applicable to most people with chronic constipation. Studies diagnosed constipation using the Rome IV criteria. Outcome was assessed as proportion of people with improvement in constipation symptoms.	Adverse events, including gastrointestinal symptoms, were reported. In one study, half of the participants in both the intervention and placebo groups required a dose reduction due to abdominal pain or diarrhoea.	Large (RR: 3.32, 95% CI: 1.59, 6.92).	Easy and convenient to take. Some patients may not like using supplements.	Inexpensive
14. Magnesium oxide supplements increase stool frequency and soften stool consistency in constipation.	Moderate	All downgraded due to imprecision.	Strong	Applicable to most people with chronic constipation. Studies diagnosed constipation using the Rome IV criteria.	Adverse events, including gastrointestinal symptoms, were reported. In one study, half of the participants in both the intervention and placebo groups required a dose reduction due to abdominal pain or diarrhoea.	Stool frequency: Large and clinically meaningful (MD: +3.72 CSBM/wk, 95% CI: 1.41, 6.03). Stool consistency: Large and clinically meaningful (MD: +1.14 points on Bristol Stool Form Scale, 95% CI: 0.48, 1.79).		
15. Magnesium oxide supplements improve global symptoms, as well as the severity of straining and the sense of incomplete evacuation in constipation.	Low	Global symptoms and incomplete evacuation: Downgraded due to imprecision. Straining: Downgraded due to inconsistency and imprecision.	Strong	Applicable to most people with chronic constipation. Studies diagnosed constipation using the Rome IV criteria.	Adverse events, including gastrointestinal symptoms, were reported. In one study, half of the participants in both the intervention and placebo groups required a dose reduction due to abdominal pain or diarrhoea.	Global symptoms: Large (MD: +3.22 points in CCCS questionnaire, 95% CI: 1.78, 4.66). Straining (severity): Large (MD: −1.09 points on 5‐point Likert scale, 95% CI: −1.64, −0.54). Incomplete evacuation (severity): Moderate (MD: −0.93 points on 5‐point Likert scale, 95% CI: −1.23, −0.63).		
16. Magnesium oxide supplements reduce the severity of bloating and abdominal discomfort in constipation.	Low	Bloating: Downgraded due to imprecision. Abdominal discomfort: Downgraded due to inconsistency and imprecision.	Strong	Applicable to most people with chronic constipation. Studies diagnosed constipation using the Rome IV criteria.	Adverse events, including gastrointestinal symptoms, were reported. In one study, half of the participants in both the intervention and placebo groups required a dose reduction due to abdominal pain or diarrhoea.	Bloating (severity): Moderate (MD: −0.81 on 5‐point Likert scale, 95% CI: −1.16, −0.46). Abdominal discomfort (severity): Moderate (MD: −0.59 on 5‐point Likert scale, 95% CI: −1.09, −0.10).		
17. Magnesium oxide supplements improve global and specific components of quality of life in constipation.	Moderate	All downgraded due to imprecision.	Strong	Applicable to most people with chronic constipation. Studies diagnosed constipation using the Rome IV criteria.	Adverse events, including gastrointestinal symptoms, were reported. In one study, half of the participants in both the intervention and placebo groups required a dose reduction due to abdominal pain or diarrhoea.	Global: Large (MD: 16.25 points on PAC‐QoL, 95% CI: 11.47–21.04). Physical: Moderate (MD: 2.60 points on PAC‐QoL, 95% CI: 1.47, 3.72). Psychosocial: Moderate (MD: 1.83 points on PAC‐QoL, 95% CI: 0.48, 3.18). Worries and concerns: Large (MD: 5.16 points on PAC‐QoL, 95% CI: 2.85, 7.46). Satisfaction: Large (MD: 6.70 points on PAC‐QoL, 95% CI: 5.09, 8.32).		
*Senna supplements*
18. Senna supplements do not impact number of people with constipation who have a clinical benefit.	Low	Downgraded due to inconsistency and imprecision.	Qualified	Applicable to most people with chronic constipation. Studies diagnosed constipation using the Rome III and IV criteria. Outcome was assessed as proportion of people with improvement in constipation symptoms, or increase of ≥ 1 complete spontaneous bowel movement/week.	Adverse events, including gastrointestinal symptoms, were reported. In one study, 83% of participants in the senna group and 50% in the placebo group required a dose reduction due to abdominal pain or diarrhoea.	No effect (RR: 2.78, 95% CI: 0.93, 8.27).	Easy and convenient to take. Some patients may not like using supplements.	Inexpensive
19. Senna supplements do not impact stool frequency in constipation.	Low	Downgraded due to inconsistency and imprecision.	Qualified	Applicable to most people with chronic constipation. Studies diagnosed constipation using the Rome III and IV criteria. Outcome was assessed as proportion of people with improvement in constipation symptoms, or increase of ≥ 1 complete spontaneous bowel movement/week.	Adverse events, including gastrointestinal symptoms, were reported. In one study, 83% of participants in the senna group and 50% in the placebo group required a dose reduction due to abdominal pain or diarrhoea.	No effect (MD: 4.20 complete spontaneous bowel movements/week, 95% CI: −2.51, 10.92).		
*Kiwifruit supplements*
20. Kiwifruit supplements do not impact stool frequency or stool consistency in constipation.	Low	All downgraded due to inconsistency and imprecision.	Strong	Applicable to most people with chronic constipation. Stool frequency and stool consistency: Studies diagnosed constipation using the Rome III criteria and presence of constipation symptoms (including infrequent bowel movements).	Adverse events, including gastrointestinal symptoms, were reported in one study.	Stool frequency: No effect (MD: 0.24 bowel movements/week, 95% CI: −0.32, 0.80). Stool consistency: No effect (MD: −0.11 points on Bristol Stool Form Scale, 95% CI: −0.31, 0.09).	Easy and convenient to take. Not widely available. Some patients may not like using supplements.	Inexpensive
21. Kiwifruit supplements improve specific symptoms of constipation only.	Very low	All downgraded due to high risk of bias, inconsistency, and imprecision.	Qualified	Applicable to most people with chronic constipation. Straining, incomplete evacuation, manual manoeuvres (frequency): Studies diagnosed constipation using the Rome III criteria.	Adverse events, including gastrointestinal symptoms, were reported in one study.	Straining (frequency): no effect (MD: −0.14 bowel movements with straining/week, 95% CI: −0.54, 0.26). Incomplete evacuation (frequency): small (MD: −0.12 bowel movements with incomplete evacuation/week, 95% CI: −0.20, −0.04). Manual manoeuvres (frequency): no effect (MD: −0.07 bowel movements with manual manoeuvres /week, 95% CI: −0.15, 0.01).		
22. Kiwifruit supplements reduce the frequency of abdominal pain in constipation, but not flatulence or bloating.	Very low	Abdominal pain and bloating: Downgraded due to high risk of bias and imprecision. Flatulence: Downgraded due to high risk of bias, inconsistency, and imprecision.	Qualified	Applicable to most people with chronic constipation. Abdominal pain, flatulence, bloating (frequency): Studies diagnosed constipation using the Rome III criteria.	Adverse events, including gastrointestinal symptoms, were reported in one study.	Abdominal pain (frequency): small (MD: −0.14 bowel movements with abdominal pain/week, 95% CI: −0.19, −0.09). Flatulence (frequency): no effect (MD: −0.11 bowel movements with flatulence/week, 95% CI: −0.26, 0.04). Bloating (frequency): no effect (MD: 0.04 bowel movements with bloating/week, 95% CI: −0.05, 0.13).		
*FOODS AND DRINKS*								
*Kiwifruits and prunes*								
23. There is no difference between kiwifruit and psyllium in the number of people with constipation who have a clinical benefit.	Low	Downgraded due to high risk of bias and imprecision.	Qualified	Applicable to most people with chronic constipation. Studies diagnosed constipation using the Rome III criteria. One study also included IBS‐C diagnosed using the Rome III criteria. Outcome was assessed as proportion of people with ≥ 1 CSBM/week, ≥ 1.5 CSBM/week, or no longer meeting the Rome III criteria.	Adverse events of gastrointestinal symptoms have been reported in one study, though these were fewer than the psyllium group (control).	No difference between groups (RR: 1.32, 95% CI: 0.91, 1.92).	Easy and convenient to eat. Widely available. Some patients may not like the taste or mouth feel.	Inexpensive
24. Kiwifruit is more effective at increasing stool frequency, but not improving stool consistency, compared with psyllium, in constipation.	Low	All downgraded due to high risk of bias and imprecision.	Qualified	Applicable to most people with chronic constipation. Stool frequency and consistency: Studies diagnosed constipation using the Rome III or IV criteria. One study also included IBS‐C diagnosed using the Rome III criteria.	Adverse events of gastrointestinal symptoms have been reported in one study, though these were fewer than the psyllium group (control).	Stool frequency: Small (MD: 0.36 CSBM/week, 95% CI: 0.24, 0.48). Stool consistency: no effect (MD: 0.32 points on Bristol Stool Form Scale, 95% CI: −0.12, 0.76).		
25. There is no difference between prunes and psyllium on stool consistency in constipation.	Very low	Downgraded due to high risk of bias and imprecision.	Qualified	Applicable to most people with chronic constipation. Stool consistency: Studies diagnosed constipation using the Rome III or IV criteria. One study also included IBS‐C diagnosed using the Rome IV criteria.	Adverse events of gastrointestinal symptoms have been reported in one study, though these were fewer than the psyllium group (control).	No difference between groups (MD: 0.45 points on Bristol Stool Form Scale, 95% CI: −0.24, 1.14).	Easy and convenient to eat. Widely available. Some patients may not like the taste.	
26. There is no difference between kiwifruits and psyllium on frequency of straining in constipation.	Very low	Downgraded due to high risk of bias, inconsistency, and imprecision.	Qualified	Applicable to most people with chronic constipation. Studies diagnosed constipation using the Rome III or IV criteria. One study also included IBS‐C diagnosed using the Rome III criteria.	Adverse events of gastrointestinal symptoms have been reported in one study, though these were fewer than the psyllium group (control).	No difference between groups (SMD: −1.62, 95% CI: −4.45, 1.21).	Easy and convenient to eat. Widely available. Some patients may not like the taste or mouth feel.	
27. There is no difference between prunes and psyllium on severity of straining in constipation.	Very low	Downgraded due to high risk of bias and imprecision.	Qualified	Applicable to most people with chronic constipation. Studies diagnosed constipation using the Rome III or IV criteria. One study also included IBS‐C diagnosed using the Rome IV criteria.	Adverse events of gastrointestinal symptoms have been reported in one study, though these were fewer than the psyllium group (control).	No difference between groups (SMD: −0.13, 95% CI: −0.69, 0.43).	Easy and convenient to eat. Widely available. Some patients may not like the taste.	
*Rye bread*
28. Rye bread is more effective than white bread at increasing stool frequency in constipation.	Low	Downgraded due to high risk of bias and imprecision.	Qualified	Applicable to most people with chronic constipation. Studies diagnosed constipation using the self‐reported constipation and presence of specific symptoms (infrequent bowel movements and/or straining).	Adverse events of gastrointestinal symptoms have been reported in one study, and these were higher compared to white bread (control). These were particularly evident in the beginning of the intervention, though they remained higher than control for the whole study duration.	Small (MD: 0.43 bowel movements/week, 95% CI: 0.03, 0.83).	Widely available. Some patients may not like rye bread. Dose required (6–7 slices) is too high and may not be realistic or manageable for patients.	Inexpensive
29. Rye bread worsens global symptoms of constipation compared to white bread.	Low	Downgraded due to high risk of bias and imprecision.	Qualified	Applicable to most people with chronic constipation. Studies diagnosed constipation using the self‐reported constipation and presence of specific symptoms (infrequent bowel movements and/or straining).	Adverse events of gastrointestinal symptoms have been reported in one study, and these were higher compared to white bread (control). These were particularly evident in the beginning of the intervention, though they remained higher than control for the whole study duration.	Small (MD: 2.00 points in scale ranging from 0 to 18, 95% CI: 0.48, 3.53).		
*High mineral content water*
30. High mineral‐content water is more effective than low mineral‐content water at increasing the number of people with constipation who have a clinical benefit.	Low	Downgraded due to high risk of bias and imprecision.	Strong	Applicable to most people with chronic constipation. Studies diagnosed constipation using the Rome III and/or presence of specific symptoms (infrequent bowel movements). Outcome was assessed as proportion of people with softer stools, or ≥ 4 bowel movements/week, or increase of ≥ 2 bowel movements/week and < 25% hard stools.	Adverse events of gastrointestinal symptoms have been reported in one study.	Moderate (RR: 1.47, 95% CI: 1.20, 1.81).	Easy and convenient to take. May be challenging to find a water high in mineral content required for laxative effect.	Inexpensive
31. There is no difference between high mineral‐content water compared to low mineral‐content water on stool frequency in constipation.	Low	Downgraded due to high risk of bias and inconsistency.	Strong	Applicable to most people with chronic constipation. Studies diagnosed constipation using the Rome III and/or presence of specific symptoms (infrequent bowel movements).	Adverse events of gastrointestinal symptoms have been reported in one study.	No effect (MD: 0.41 bowel movements/week, 95% CI: −0.05, 0.88).		
32. There is no difference between high mineral‐content water and low mineral‐content water on global symptoms of constipation.	Moderate	Downgraded due to imprecision.	Strong	Applicable to most people with chronic constipation. Studies diagnosed constipation using the Rome III and/or presence of specific symptoms (infrequent bowel movements).	Adverse events of gastrointestinal symptoms have been reported in one study.	No effect (SMD: −0.04, −0.27, 0.18).		
33. There is no difference between high mineral‐content water and low mineral‐content water on the severity of abdominal pain in constipation.	Moderate	Downgraded due to high risk of bias.	Strong	Applicable to most people with chronic constipation. Studies diagnosed constipation using the Rome III and/or presence of specific symptoms (infrequent bowel movements).	Adverse events of gastrointestinal symptoms have been reported in one study.	No effect (SMD: −0.17, 95% CI: −0.42, 0.08).		
34. There is no difference between high mineral‐content water and low mineral‐content water on global quality of life in constipation.	Low	Downgraded due to high risk of bias and imprecision.	Strong	Applicable to most people with chronic constipation. Studies diagnosed constipation using the Rome III and/or presence of specific symptoms (infrequent bowel movements).	Adverse events of gastrointestinal symptoms have been reported in one study.	No effect (SMD: 0.13, 95% CI: −0.10, 0.36).		

### 3.1 Fibre Supplements

Statements on fibre supplements were derived from the evidence generated in a systematic review and meta‐analysis of 16 RCTs that included 1251 people with chronic constipation and investigated the effect of various fibre supplements on chronic constipation [12].

**Fibre supplements and response to treatment**

1. **Specific types of fibre supplements increase the number of people with constipation who have a clinical benefit.**
*Level of evidence: low* 
*Strength of recommendation: strong*
  1.1. **Psyllium supplements increase the number of people with constipation who have a clinical benefit.**
*Level of evidence: low*
*Strength of recommendation: strong*
  1.2. **Polydextrose, mixtures of inulin and other fibres, and galacto‐oligosaccharide supplements do not impact the number of people with constipation who have a clinical benefit.**
*Level of evidence: low*
*Strength of recommendation: strong*

Nine RCTs, including 802 people with chronic constipation, assessed the effect of fibre supplements on symptomatic response to treatment, measured as a binary outcome [12]. Overall, fibre supplementation increased the likelihood of a beneficial response to treatment by 48% compared to placebo (RR: 1.48, 95% CI: 1.17, 1.88). When performing subgroup analyses based on the specific type of fibre used, three RCTs investigated the effect of psyllium (doses 10.8, 20 and 40 g/d) on response to treatment and reported a significant improvement, compared to placebo (RR: 1.82, 95% CI: 1.51, 2.20). However, subgroup analyses of polydextrose, galacto‐oligosaccharides and mixtures of inulin and other fibres had no impact. Pectin and wheat bran were only investigated in one RCT, and thus, no statement could be developed for these fibre supplements. Higher doses of fibre (> 10 g/d) led to a significantly higher response to treatment (RR: 1.72, 95% CI: 1.35, 2.18), whereas lower doses of fibre (≤ 10 g/d) did not (RR: 1.01, 95% CI: 0.6, 1.34). All treatment durations (less than or over 4 weeks) were effective at increasing response to treatment.

**Fibre supplements and stool output**

2. **Specific types of fibre supplements increase stool frequency and soften stool consistency in constipation.**
*Level of evidence: low*
*of recommendation: strong*
  2.1. **Psyllium supplements increase stool frequency and soften stool consistency in constipation.**
*Level of evidence: low*
*Strength of recommendation: strong*
  2.2. **Inulin‐type fructan supplements do not impact stool frequency in constipation, but soften stool consistency.**
*Level of evidence: low*
*Strength of recommendation: qualified*
  2.3. **Mixtures of inulin and other fibre supplements do not impact stool frequency in constipation**
*Level of evidence: very low*
*Strength of recommendation: strong*
  2.4. **Polydextrose and galacto‐oligosaccharide supplements do not impact stool frequency or stool consistency in constipation.**
*Level of evidence: low*
*Strength of recommendation: strong*

Fourteen RCTs, including 1040 people with chronic constipation, investigated the effect of fibre supplements on stool frequency and were meta‐analysed [12]. Overall, fibre supplements significantly increased stool frequency, with a moderate to large effect, compared to placebo (SMD: 0.72, 95% CI: 0.36, 1.08). When performing subgroup analyses based on the specific type of fibre used, three RCTs investigated the effect of psyllium on stool frequency and showed a significant increase in stool frequency (SMD: 1.13, 95% CI: 0.39, 1.88). However, subgroup analyses of polydextrose, inulin‐type fructans, galacto‐oligosaccharides, and mixtures of inulin with other fibres had no impact. Pectin, wheat bran and unspecified fibres were only investigated in one RCT, and thus, no statement could be developed for these fibre supplements. Higher doses of fibre (> 10 g/d) significantly increased stool frequency (SMD: 0.93, 95% CI: 0.49, 1.38), whereas lower doses of fibre (≤ 10 g/d) did not. Greater treatment durations of ≥ 4 weeks significantly increased stool frequency (SMD: 1.13, 95% CI: 0.50, 1.76), though shorter durations did not.

Ten RCTs, including 918 people with chronic constipation, investigated the effect of fibre supplements on stool consistency and were meta‐analysed. Overall, fibre supplements significantly softened stool consistency compared to placebo (SMD: 0.32, 95% CI: 0.18, 0.46); however, the improvement was small and unlikely to be clinically meaningful to patients. When performing subgroup analyses based on the specific type of fibre used, three RCTs investigated the effect of psyllium on stool consistency and showed it significantly softened stool consistency (SMD: 0.52, 95% CI: 0.25, 0.78). Two RCTs investigated the effect of inulin‐type fructans and showed they significantly softened stool consistency, albeit the effect size was small and unlikely to be clinically meaningful (SMD: 0.36, 95% CI: 0.03, 0.70). However, subgroup analysis of polydextrose reported no impact. Galacto‐oligosaccharides and mixtures of inulin with resistant maltodextrin were only investigated in one RCT, and thus, no statement could be developed for these fibre supplements. Higher doses of fibre (> 10 g/d) significantly softened stool consistency (SMD: 0.42, 95% CI: 0.26, 0.59), whereas lower doses of fibre did not. All treatment durations (less than or over 4 weeks) were effective at increasing response to treatment.

**Fibre supplements and symptoms**

3. **Overall, fibre supplements do not impact global symptoms of constipation, but specific types of fibre supplements improve specific symptoms of constipation.**
*Level of evidence: low*
*Strength of recommendation: qualified*
  3.1. **Psyllium supplements reduce the severity of straining in constipation.**
*Level of evidence: low*
*Strength of recommendation: strong*
  3.2. **Polydextrose supplements do not impact global symptoms or specific symptoms of constipation.**
*Level of evidence: low*
*Strength of recommendation: strong*
  3.3. **Galacto‐oligosaccharide supplements do not impact global symptoms of constipation.**
*Level of evidence: low*
*Strength of recommendation: strong*

Five RCTs, including 531 people with chronic constipation, investigated the effect of fibre supplements on global symptom scores using questionnaires [12]. Overall, fibre supplements had no impact on global symptom scores (SMD: −0.15, 95% CI: −0.39, 0.08), however, when only those that were administered for greater durations (≥ 4 weeks) were meta‐analysed, this led to significant improvements (SMD: −0.42, 95% CI: 0.77, −0.06), highlighting fibre supplements should be trialled for a minimum of 4 weeks. No dose–response effect was shown. When performing subgroup analyses based on the specific type of fibre used, polydextrose and galacto‐oligosaccharides had no impact on global symptoms. Pectin and wheat bran were only investigated in one RCT, and thus, no statement could be developed for these fibre supplements.

Four RCTs, including 489 people with chronic constipation, investigated the effect of fibre supplements on the severity of straining. Overall, fibre supplements significantly reduced straining severity, compared to control (SMD: −0.32, 95% CI: −0.59, −0.04). When performing subgroup analyses based on the specific type of fibre used, two RCTs investigated the effect of psyllium (10.2 and 19.2 g/d) and showed a significant reduction in the severity of straining (SMD: −0.65, 95% CI: −0.91, −0.39). However, subgroup analyses of polydextrose had no impact. Inulin‐type fructans were only investigated in one RCT, and thus, no statement could be developed. Higher doses (> 10 g/d) reduced straining severity (SMD: −0.45, 95% CI: −0.73, −0.16), but lower doses did not, and it is unclear which treatment duration is beneficial.

Two RCTs, including 110 people with chronic constipation, investigated the effect of fibre supplements on the severity of a sense of incomplete evacuation. Overall, fibre supplements had no impact on the severity of a sense of incomplete evacuation. It was not possible to produce statements for specific types of fibre (psyllium and inulin‐type fructans), as they were only investigated in one RCT each.

**Fibre supplements and adverse events**

4. **Specific types of fibre supplements increase the severity of flatulence in constipation, but not bloating or abdominal pain/discomfort.**
*Level of evidence: low*
*Strength of recommendation: strong*
  4.1. **Inulin‐type fructan supplements increase the severity of flatulence in constipation, but not bloating.**
*Level of evidence: low*
*Strength of recommendation: strong*

Three RCTs, including 153 people with chronic constipation, investigated the effect of fibre supplements on the severity of flatulence [12]. Overall, fibre supplements significantly worsened flatulence severity, compared to placebo (SMD: 0.80, 95% CI: 0.47, 1.13). When performing subgroup analyses based on the specific type of fibre used, two RCTs investigated the effect of inulin‐type fructans (12 and 15 g/d) and showed a significantly worse severity of flatulence, compared to placebo (SMD: 0.79, 95% CI: 0.44, 1.14). Wheat bran was only investigated in one RCT, and thus, no statement could be developed for it.

Four RCTs, including 239 people with chronic constipation, investigated the effect of fibre supplements on the severity of bloating. Overall, fibre supplements had no impact on the severity of bloating, compared to placebo (SMD: 0.07, 95% CI: −0.38, 0.51). When performing subgroup analyses based on the specific type of fibre used, two RCTs investigated the effect of inulin‐type fructans and showed no impact on the severity of bloating. Pectin and wheat bran were only investigated in one RCT, and thus, no statement could be developed for these fibre supplements.

Three RCTs, including 299 people with chronic constipation, investigated the effect of fibre supplements on the severity of abdominal pain/discomfort. Overall, fibre supplements had no impact on the severity of abdominal pain/discomfort (SMD: −0.14, 95% CI: −0.36, 0.09). It was not possible to produce statements for specific types of fibre (psyllium, inulin‐type fructans and wheat bran), as they were only investigated in one RCT each.

**Fibre supplements and quality of life**

5. **Polydextrose supplements do not impact global or specific components of quality of life in constipation.**
*Level of evidence: moderate*
*Strength of recommendation: strong*

Two RCTs, including 296 people with chronic constipation, investigated the effect of polydextrose supplements on global and specific components of QoL [12]. Polydextrose supplements had no impact on global QoL (MD: −0.04 scale points, 95% CI: −0.19, 0.10 scale points; higher score is more severe), nor on the QoL components of satisfaction, physical discomfort, worries and concerns, or psychosocial discomfort.

**Fibre supplements: Good Practice statements**

- Fibre supplement doses above 10 g/d are optimal for increasing the number of people with constipation who have a clinical benefit, improving stool output and reducing the severity of straining (evidence‐based recommendation).
- Consuming fibre supplements for a minimum duration of 4 weeks is optimal for increasing stool frequency and improving global constipation symptoms of constipation (evidence‐based recommendation).
- In people with constipation who experience tolerance issues with fibre, fibre supplement intake may be increased gradually with weekly increments to avoid adverse effects, such as bloating and flatulence (expert opinion recommendation).
- When advising the use of inulin‐type fructan supplements in constipation, the possibility of increased flatulence should be discussed (evidence‐based recommendation).
- Fibre supplements should be accompanied by additional fluid intake where clinically appropriate (expert opinion recommendation).

### 3.2 Probiotic Supplements

Statements on probiotic supplements were derived from the evidence generated in a systematic review and meta‐analysis of 30 RCTs that included 2804 people with chronic constipation and investigated the effect of various probiotic supplements on chronic constipation [13].

**Probiotic supplements and response to treatment**

6. **Probiotics overall may increase the number of people with constipation who have a clinical benefit, though it is unclear which species or strains are effective.**
*Level of evidence: low*
*Strength of recommendation: qualified*
6.1. **Multi‐strain probiotic supplements,** ***Bifidobacterium lactis*** **supplements, and** ***Bacillus coagulans*** **supplements do not impact the number of people with constipation who have a clinical benefit.**
*Level of evidence: low*
*Strength of recommendation: qualified*

Fourteen RCTs, including 1214 people with chronic constipation, assessed the effect of various probiotic supplements in symptomatic response to treatment, measured as a binary outcome [13]. Overall, probiotic supplements increased the likelihood of a beneficial response to treatment by 28%, compared to placebo (RR: 1.28, 95% CI: 1.07, 1.52). However, when performing subgroup analyses based on the specific probiotic strain or species, neither multi‐strain probiotics, *Bifidobacterium lactis* nor *Bacillus coagulans* significantly increased response to treatment, compared to placebo. *Bifidobacterium bifidum* CCFM 16, *Lactobacillus casei* Shirota, *Lactobacillus paracasei* IMPC 2.1 and *Lactobacillus reuteri* DSM 17938 were only investigated in one RCT and thus, no statement could be developed for these strains of probiotic supplements. Therefore, while overall probiotics were found to improve response to treatment, when meta‐analysed altogether, no single probiotic species or strain was found to be effective in increasing response to treatment, and thus, no specific probiotic species or strain recommendations can be made for this outcome. No subgroup differences were found on the dose (< or ≥ 10^10^ CFU/d) or treatment duration (< or ≥ 4 weeks).

**Probiotic supplements and stool output**

7. **Probiotics overall, and some specific species, increase stool frequency, but do not impact stool consistency in constipation.**
*Level of evidence: low*
*Strength of recommendation: qualified*
  7.1. **Multi‐strain probiotic supplements do not impact stool frequency in constipation, but soften stool consistency.**
*Level of evidence: low*
*Strength of recommendation: qualified*
  7.2. ***Bifidobacterium lactis*** **supplements increase stool frequency in constipation, but do not impact stool consistency.**
*Level of evidence: moderate*
*Strength of recommendation: qualified*
  7.3. ***Bacillus coagulans*** **Unique IS2 supplements do not impact stool frequency or stool consistency in constipation.**
*Level of evidence: low*
*Strength of recommendation: qualified*
  7.4. ***Lactobacillus casei*** **Shirota supplements do not impact stool frequency or stool consistency in constipation.**
*Level of evidence: very low*
*Strength of recommendation: strong*

Nineteen RCTs, including 1989 people with chronic constipation, assessed the effect of various probiotic supplements on stool frequency [13]. Overall, probiotic supplements significantly increased stool frequency, compared to placebo (SMD: 0.71, 95% CI: 0.37, 1.04). When performing subgroup analyses based on the specific probiotic strain or species, seven RCTs investigated the effect of *B. lactis* and showed a significant increase in stool frequency, of small to moderate effect size, compared to placebo (SMD: 0.48, 95% CI: 0.19, 0.77). The *Bifidobacterium lactis* strains investigated included *B. lactis* LMG P‐21384, *B. lactis* NCC2818, *B. lactis* Bi‐07, *B. lactis* DN‐173 010, *Bacillus lactis* HN019, *B. lactis* GCL2505 and *B. lactis* MN‐gup. Subgroup analyses of multi‐strain probiotics, *Bacillus coagulans* Unique IS2 and *L. casei* Shirota supplements showed no impact on stool frequency. *Bifidobacterium animalis*, *Bacillus coagulans* lilac 01, *L. reuter*i DSM 17938 and *E. coli* Nissle 1917 were only investigated in one RCT, and thus, no statement could be developed for these probiotic supplements. No subgroup differences were found on the dose (< or ≥ 10^10^ CFU/d) or treatment duration (< or ≥ 4 weeks).

Sixteen RCTs, including 1772 people with chronic constipation, assessed the effect of various probiotic supplements on stool consistency [13]. Overall, probiotic supplements did not impact stool consistency, compared to placebo (SMD: 0.26, 95% CI: −0.03, 0.54). When performing subgroup analyses based on the specific probiotic species or strain, three RCTs investigated the effect of multi‐strain probiotics and showed they significantly softened stool consistency, albeit with a small effect size (SMD: 0.27, 95% CI: 0.03, 0.51). Subgroup analyses of *B. lactis, Bacillus coagulans* Unique IS2 and *L. casei* Shirota showed no impact on stool consistency. *Bifidobacterium animalis, B. longum* BB536, *Bacillus coagulans* lilac‐01 and *L. paracasei* IMPC 2.1 were only investigated in one RCT, and thus, no statement could be developed for these probiotic supplements. Though there is sufficient evidence to recommend multi‐strain probiotics for softening stool consistency, there is insufficient evidence to make recommendations on strain‐specific preparations. No subgroup differences were found on the dose (< or ≥ 10^10^ CFU/d) or treatment duration (< or ≥ 4 weeks).

**Probiotic supplements and symptoms**

8. **Probiotics overall improve global symptoms of constipation, but not the majority of individual symptoms, though it is unclear which species or strains are effective.**
*Level of evidence: very low*
*Strength of recommendation: qualified*
  8.1. **Multi‐strain probiotic supplements do not impact global symptoms of constipation.**
*Level of evidence: low*
*Strength of recommendation: qualified*
  8.2. ***Bifidobacterium lactis*** **supplements do not impact global symptoms or specific symptoms of constipation.**
*Level of evidence: low*
*Strength of recommendation: qualified*
  8.3. ***B. coagulans*** **Unique IS2 supplements improve specific symptoms of constipation.**
*Level of evidence: low*
*Strength of recommendation: qualified*
  8.4. ***Lactobacillus casei*** **Shirota supplements do not impact global symptoms of constipation.**
*Level of evidence: very low*
*Strength of recommendation: strong*

Eight RCTs, including 915 people with chronic constipation, investigated the effect of various probiotic supplements on global symptom scores using questionnaires [13]. Overall, probiotic supplements significantly reduced global symptoms, with a small to moderate effect size, compared to placebo (SMD: −0.46, 95% CI: −0.89, −0.04). However, when performing subgroup analyses based on the specific probiotic species or strain, neither the *B. lactis* species nor the multi‐strain probiotic supplements had an impact on global symptoms. *B. longum* BB536, *L. casei* Shirota and *L. paracasei* IMPC 2.1 were only investigated in one RCT each, and thus, no statement on global symptoms could be developed for these probiotic supplements. No subgroup differences were found on the dose (< or ≥ 10^10^ CFU/d) or treatment duration (< or ≥ 4 weeks).

Three RCTs, including 409 people with chronic constipation, investigated the effect of various probiotic supplements on the severity of straining [13]. Overall, probiotic supplements had no impact on the severity of straining compared to placebo (SMD: −0.11, 95% CI: −0.32, 0.10). Similarly, when performing subgroup analyses based on the specific probiotic strain or species, *B. lactis* had no impact on the severity of straining. *Bacillus coagulans* lilac‐01 was only investigated in one RCT, and thus, no statement on the severity of straining could be developed for it.

Two RCTs, including 79 people with chronic constipation, investigated the effect of various probiotic supplements on the frequency of straining [13]. Overall, probiotic supplements had no impact on the frequency of straining compared to placebo (SMD: −0.48, 95% CI: −1.48, 0.52). *Bifidobacterium lactis* Bi‐07 and *B. animalis* were only investigated in one RCT each, and thus, no statement on the frequency of straining could be developed for these.

Five RCTs, including 588 people with chronic constipation, investigated the effect of various probiotic supplements on the severity of a sense of incomplete evacuation [13]. Overall, probiotics reduced the severity of a sense of incomplete evacuation, with a large effect size, compared to placebo (SMD: −0.81, 95% CI: −1.17, −0.45). However, when performing subgroup analyses based on the specific probiotic species or strain, *Bifidobacterium lactis* had no impact on the severity of a sense of incomplete evacuation. Multi‐strain probiotics, *Bacillus coagulans* lilac‐01, *L. casei* Shirota, and *L. paracasei* IMPC 2.1 were only investigated in one RCT, and thus, no statement could be developed for these probiotic supplements. All treatment durations were effective in reducing the severity of a sense of incomplete evacuation, although shorter treatment durations (< 4 weeks) led to a greater effect in reducing the severity of a sense of incomplete evacuation (SMD: −1.46, 95% CI: −2.36, −0.55; subgroup *p*‐value = 0.07) than longer durations.

Four RCTs, including 219 people with chronic constipation, investigated the effect of various probiotic supplements on the frequency of a sense of incomplete evacuation [13]. Overall, probiotic supplements had no impact on the frequency of a sense of incomplete evacuation compared to placebo (SMD: −0.41, 95% CI: −1.18, 0.36). Similarly, when performing subgroup analyses based on the specific probiotic species or strain, *Bacillus coagulans* Unique IS2 had no impact on the frequency of a sense of incomplete evacuation. *Bifidobacterium lactis* Bi‐07 and *B. animalis* were only investigated in one RCT each, and thus, no statement could be developed for these.

Two RCTs, including 90 people with chronic constipation, investigated the effect of various probiotic supplements on the frequency of use of manual manoeuvres (e.g., anal digitation) and the frequency of sensation of anorectal obstruction [13]. Overall, probiotic supplements had no impact on the frequency of use of manual manoeuvres (SMD: −0.26, 95% CI: −0.82, 0.30) nor the frequency of sensation of anorectal obstruction (SMD: −0.31, 95% CI: −1.13, 0.51). *Bifidobacterium lactis* Bi‐07 and *B. animalis* were only investigated in one RCT each, and thus, no statement could be developed for these outcomes.

Two RCTs, including 200 people with chronic constipation, investigated the effect of *Bacillus coagulans* Unique IS2 supplements on the frequency of defecation pain and found that it reduced the frequency of defecation pain (MD: −0.64 points, 95% CI: −1.27, −0.02; lower score denotes less frequent symptoms) [13].

**Probiotic supplements and adverse events**

9. **Probiotics overall reduce the severity of flatulence in constipation, but do not impact abdominal pain/discomfort or bloating, though it is unclear which species or strains are effective.**
*Level of evidence: low*
*Strength of recommendation: qualified*
  9.1. **Multi‐strain probiotic supplements do not impact the severity of flatulence, abdominal pain/discomfort or bloating in constipation.**
*Level of evidence: very low*
*Strength of recommendation: qualified*
  9.2. ***Bifidobacterium lactis*** **supplements do not impact the severity of abdominal pain or bloating in constipation.**
*Level of evidence: very low*
*Strength of recommendation: qualified*
  9.3. ***Bacillus coagulans*** **Unique IS‐2 supplements decrease frequency of abdominal pain in constipation.**
*Level of evidence: Low*
*Strength of recommendation: qualified*
  9.4. ***Lactobacillus casei*** **Shirota supplements do not impact the severity of bloating in constipation.**
*Level of evidence: low*
*Strength of recommendation: strong*

Five RCTs, including 821 people with chronic constipation, investigated the effect of various probiotic supplements on the severity of bloating [13]. Overall, probiotics did not impact the severity of bloating, compared to placebo (SMD: −0.38, 95% CI: −0.82, 0.06). Similarly, when performing subgroup analyses based on the specific probiotic strain or species, multi‐strain probiotics, *B. lactis* and *L. casei* Shirota had no impact on the severity of bloating.

Three RCTs, including 299 people with chronic constipation, investigated the effect of various probiotic supplements on the severity of flatulence [13]. Overall, probiotics led to a marginally significant reduction in the severity of flatulence, compared to placebo (SMD: −0.37, 95% CI: −0.73, −0.00). However, when performing subgroup analyses based on the specific probiotic species or strain, multi‐strain probiotics had no impact on the severity of flatulence. *L. casei* Shirota was only investigated in one RCT, and thus, no statement could be developed for it.

Three studies, including 497 people with chronic constipation, investigated the effect of various probiotic supplements on the severity of abdominal pain [13]. Overall, probiotic supplements had no impact on the severity of abdominal pain compared to control (SMD: −0.10, 95% CI: 0.28, 0.09). Similarly, when performing subgroup analyses based on the specific probiotic strain or species, multi‐strain probiotics and *B. lactis* HN019 had no impact on this outcome.

Two RCTs, including 200 people with chronic constipation, investigated the effect of *Bacillus coagulans* Unique IS2 supplements on frequency of abdominal pain and showed a significant reduction in frequency of abdominal pain (MD: −0.67 points, 95% CI: −1.14, −0.20; lower score, less frequent symptoms) [13].

**Probiotic supplements and quality of life**

10. **Probiotics overall do not impact global or specific components of quality of life in constipation.**
*Level of evidence: low*
*Strength of recommendation: qualified*
  10.1. **Multi‐strain probiotic supplements do not impact global quality of life in constipation, but improve specific components of quality of life.**
*Level of evidence: low*
*Strength of recommendation: qualified*
  10.2. ***Bifidobacterium lactis*** **supplements do not impact global or specific components of quality of life in constipation.**
*Level of evidence: moderate*
*Strength of recommendation: qualified*

Six RCTs, including 755 people with chronic constipation, investigated the effect of various probiotic supplements on global QoL [13]. Overall, probiotic supplements had no impact on global QoL (MD: −0.13, 95% CI: −0.36, 0.10; higher score, better QoL), nor on specific components of QoL (physical discomfort, psychosocial discomfort, worries and concern, satisfaction), compared to placebo. Similarly, when performing subgroup analyses based on the specific probiotic species or strain, multi‐strain probiotics and *B. lactis* supplements had no impact on global QoL, nor on specific components of QoL, except for multi‐strain probiotic supplements, which significantly improved the worries and concerns and satisfaction components of QoL. *Bifidobacterium bifidum* was only investigated in one RCT for global QoL, and thus, no statement could be developed for it.

**Probiotic supplements: Good Practice statements**

Though some species and strains of probiotics, such as *B. lactis* and *Bacillus coagulans* Unique IS2, may improve constipation, in general, there is a lack of convincing evidence to recommend specific strains of probiotics in constipation (evidence‐based recommendation). Therefore, clinicians may support patients who wish to try probiotics, and advise them to try a probiotic brand of their choice for at least 4 weeks, following the instructions recommended by the manufacturer (expert opinion recommendation).

### 3.3 Synbiotic Supplements

Recommendation statements on synbiotic supplements were derived from the evidence generated in a systematic review and meta‐analysis of three RCTs that included 275 people with chronic constipation and investigated the effect of various synbiotic supplements on chronic constipation [13]. Statements for stool frequency, stool consistency and global symptoms of constipation were developed only, as only these outcomes were reported in two or more RCTs.

**Synbiotic supplements and stool output**

11. **Synbiotics do not impact stool frequency or stool consistency in constipation.**
*Level of evidence: low*
*Strength of recommendation: qualified*

Three RCTs, including 202 people with chronic constipation, investigated the effect of various combinations of probiotic and prebiotic (synbiotic) supplements on stool frequency and stool consistency [13]. Overall, synbiotic supplements had no impact on stool frequency (MD: 0.54 bowel movements/week, 95% CI: −0.80, 1.87) or stool consistency (SMD: 0.16, 95% CI: −0.48, 0.81) compared to placebo.

**Synbiotic supplements and symptoms**

12. **Synbiotics do not impact global symptoms of constipation.**
*Level of evidence: very low*
*Strength of recommendation: qualified*

Two RCTs, including 176 people with chronic constipation, investigated the effect of various combinations of probiotic and prebiotic (synbiotic) supplements on global symptoms of constipation [13]. Overall, synbiotic supplements had no impact on global symptoms of constipation (SMD: −0.55, 95% CI: −1.57, 0.48).

## 4 Food Supplements

Recommendation statements were generated for magnesium oxide supplements, senna supplements, and kiwifruit supplements. No statements were generated for any vitamin supplements due to a lack of evidence.

### 4.1 Magnesium Oxide Supplements

Statements on magnesium oxide supplements were derived from the evidence generated in a systematic review and meta‐analysis of 2 RCTs that included 94 people with chronic constipation and investigated the effect of 1.5 g/d of magnesium oxide supplements for 4 weeks [11].

**Magnesium oxide supplements and response to treatment**

13. **Magnesium oxide supplements increase the number of people with constipation who have a clinical benefit.**
*Level of evidence: moderate*
*Strength of recommendation: strong*

Two RCTs, including 94 people with chronic constipation, investigated the effect of magnesium oxide supplements on symptomatic response to treatment, measured as a binary outcome [11]. Overall, magnesium oxide supplements increased the likelihood of a beneficial response to treatment by 232% compared to control (RR: 3.32, 95% CI: 1.59, 6.92).

**Magnesium oxide supplements and stool output**

14. **Magnesium oxide supplements increase stool frequency and soften stool consistency in constipation.**
*Level of evidence: moderate*
*Strength of recommendation: strong*

Two RCTs, including 93 people with chronic constipation, investigated the effect of magnesium oxide supplements on stool frequency and consistency compared to control [11]. Overall, magnesium oxide supplements significantly increased stool frequency by 3.7 complete spontaneous bowel movements/week compared to control (95% CI: 1.41, 6.03). Magnesium oxide supplements also significantly softened stool consistency as evidenced by a 1.14 point (95% CI: 0.42, 1.79) increase in the Bristol Stool Form Scale. The magnitude of these effect sizes is large and clinically meaningful.

**Magnesium oxide supplements and symptoms**

15. **Magnesium oxide supplements improve global symptoms, as well as the severity of straining and the sense of incomplete evacuation in constipation.**
*Level of evidence: low*
*Strength of recommendation: strong*

Two RCTs, including 93 people with chronic constipation, investigated the effect of magnesium oxide supplements on global symptoms of constipation using the Constipation Scoring System questionnaire, which provides scores ranging from 0 to 30 (higher score, worse symptoms) [11]. Overall, magnesium oxide supplements significantly reduced global symptoms of constipation by −3.2 points, compared to control (95% CI: 1.8, 4.7 points).

Two RCTs, including 93 people with chronic constipation, investigated the effect of magnesium oxide supplements on the severity of straining and a sense of incomplete evacuation using a 5‐point scale (higher score, worse symptoms) [11]. Overall, magnesium oxide supplements significantly reduced the severity of both straining (MD: −1.1, 95% CI: −1.6, −0.5) and a sense of incomplete evacuation (MD: −0.9, 95% CI: −1.2, −0.6), compared to control.

**Magnesium oxide supplements and adverse events**

16. **Magnesium oxide supplements reduce the severity of bloating and abdominal discomfort in constipation.**
*Level of evidence: low*
*Strength of recommendation: strong*

Two RCTs, including 93 people with chronic constipation, investigated the effect of magnesium oxide supplements on the severity of bloating and abdominal discomfort using a 5‐point scale (higher score, worse symptoms) [11]. Overall, magnesium oxide supplements significantly reduced the severity of bloating (MD: −0.8, 95% CI: −1.2, −0.5) and abdominal discomfort (MD: −0.6, 95% CI: −1.1, −0.1), compared to control.

**Magnesium oxide supplements and quality of life**

17. **Magnesium oxide supplements improve global and specific components of quality of life in constipation.**
*Level of evidence: moderate*
*Strength of recommendation: strong*

Two RCTs, including 93 people with chronic constipation, investigated the effect of magnesium oxide supplements on global quality of life using the Patient Assessment of Constipation Quality of Life questionnaire, which provides scores ranging from 0 to 112 [11]. Overall, magnesium oxide supplements significantly improved quality of life, compared to control (MD: +16.3 points, 95% CI: 11.5, 21.0). Magnesium oxide supplements also improved all domains of quality of life assessed, including physical (MD: +2.6 points, 95% CI: 1.5, 3.7), psychosocial (MD: +1.8 points, 95% CI: 0.5, 3.2), worries and concerns (MD: +5.2 points, 95% CI: 2.9, 7.5), and satisfaction (MD: +6.7 points, 95% CI: 5.1, 8.3).

**Magnesium oxide supplements: Good Practice statements**

Magnesium oxide supplements at a dose of 0.5–1.5 g/d for at least 4 weeks may be recommended in constipation, as clinically appropriate (evidence‐based recommendation). Magnesium oxide supplements may be increased gradually with weekly increments, while monitoring tolerance, starting at a dose of 0.5 g/d (evidence‐based recommendation).

### 4.2 Senna Supplements

Statements on senna supplements were derived from the evidence generated in a systematic review and meta‐analysis of 2 RCTs that included 254 people with chronic constipation and investigated the effect of senna supplements, compared to placebo [11].

**Senna supplements and response to treatment**

18. **Senna supplements do not impact number of people with constipation who have a clinical benefit.**
*Level of evidence: low*
*of recommendation: qualified*

Two RCTs, including 254 people with chronic constipation, investigated the effect of senna supplements on symptomatic response to treatment, measured as a binary outcome [11]. Overall, senna supplements had no impact on response to treatment compared to placebo (RR: 2.78, 95% CI: 0.93, 8.27). While each RCT showed a significant improvement in response to treatment separately, when they were meta‐analysed together, there was no longer a significant impact, and there was also significant heterogeneity (*I* ^2^ = 80%, *p* = 0.03).

**Senna supplements and stool output**

19. **Senna supplements do not impact stool frequency in constipation.**
*Level of evidence: low*
*Strength of recommendation: qualified*

Two RCTs, including 254 people with chronic constipation, investigated the effect of senna supplements on stool frequency compared to placebo [11]. Overall, senna supplements had no impact on stool frequency compared to placebo (MD: 4.2 bowel movements/week, 95% CI: −2.5, 10.9). While each RCT showed a significant improvement in response to treatment separately, when they were meta‐analysed together, there was no longer a significant impact, the CI was large, and there was also significant heterogeneity (*I* ^2^ = 99%, *p* < 0.001).

### 4.3 Kiwifruit Supplements

Statements on kiwifruit supplements were derived from the evidence generated in a systematic review and meta‐analysis of 3 RCTs that included 135 people with chronic constipation and investigated the effect of kiwifruit supplements, in the form of a capsule (2 RCTs) or a sachet (1 RCT), on chronic constipation [11].

**Kiwifruit supplements and stool output**

20. **Kiwifruit supplements do not impact stool frequency or stool consistency in constipation.**
*Level of evidence: low*
*Strength of recommendation: strong*

Three RCTs, including 123 people with chronic constipation, investigated the effect of kiwifruits on stool frequency and consistency compared to control [11]. Overall, kiwifruit supplements had no impact on stool frequency (MD: 0.24 bowel movements/week, 95% CI: −0.32, 0.80) or stool consistency (MD: −0.11 points on the Bristol Stool Form Scale, 95% CI: −0.31, 0.09, *p* = 0.29).

**Kiwifruit supplements and symptoms**

21. **Kiwifruit supplements improve specific symptoms of constipation only.**
*Level of evidence: very low*
*Strength of recommendation: qualified*

One RCT that had three kiwifruit supplement arms and included 36 people with chronic constipation investigated the effect of kiwifruit supplements on the frequency of a sense of incomplete evacuation and straining, although it is unclear which assessment tool was used [11]. Overall, when these three trial arms were meta‐analysed, kiwifruit supplements significantly reduced the frequency of a sense of incomplete evacuation compared to control (MD: −0.12, 95% CI: −0.2, 0.04). However, kiwifruit supplements had no impact on frequency of straining (MD: −0.14, 95% CI: −0.54, 0.26) or manual manoeuvres to aid defaecation (MD: −0.07, 95% CI: −0.15, 0.01) compared to control.

**Kiwifruit supplements and adverse events**

22. **Kiwifruit supplements reduce the frequency of abdominal pain in constipation, but not flatulence or bloating.**
*Level of evidence: very low*
*Strength of recommendation: qualified*

One RCT that had three kiwifruit supplement arms and included 36 people with chronic constipation, investigated the effect of kiwifruit supplements on frequency of abdominal pain, flatulence and bloating, although it is unclear which assessment tool was used [11]. Overall, kiwifruit supplements significantly reduced the frequency of abdominal pain (MD: −0.14, 95% CI: −0.19, −0.09), but had no impact on flatulence (MD: −0.11, 95% CI: −0.26, 0.04) or bloating (MD: 0.04, 95% CI: −0.05, 0.13) compared to control.

## 5 Foods and Drinks

### 5.1 Fruits

Statements on fruits were derived from the evidence generated in a systematic review and meta‐analysis of 4 RCTs on kiwifruits and 2 RCTs on prunes that included a total of 258 people with chronic constipation and investigated the effect of these fruits on chronic constipation compared to psyllium supplements, a known effective intervention [14].

**Kiwifruits and response to treatment**

23. **There is no difference between kiwifruit and psyllium in the number of people with constipation who have a clinical benefit.**
*Level of evidence: low*
*Strength of recommendation: qualified*

Two RCTs, including 184 people with chronic constipation, assessed the effect of kiwifruit in symptomatic response to treatment, measured as a binary outcome [14]. Overall, kiwifruits had no greater impact on response to treatment compared to psyllium supplements (RR: 1.32, 95% CI: 0.91, 1.92).

**Kiwifruits, prunes and stool output**

24. **Kiwifruit is more effective at increasing stool frequency, but not improving stool consistency, compared with psyllium, in constipation.**
*Level of evidence: low*
*Strength of recommendation: qualified*
25. **There is no difference between prunes and psyllium on stool consistency in constipation.**
*Level of evidence: very low*
*Strength of recommendation: qualified*

Three RCTs, including 192 people with chronic constipation, assessed the effect of kiwifruit on stool frequency and consistency and were meta‐analysed [14]. Overall, kiwifruits significantly increased stool frequency by +0.36 bowel movements per week (95% CI: 0.24, 0.48 bowel movements/week), compared to psyllium supplements, albeit this effect size is small. Kiwifruits had no significantly greater impact on stool consistency when compared to psyllium supplements (MD: +0.32 points on the Bristol Stool Form Scale, 95% CI: −0.12, 0.76 points).

Two RCTs, including 86 people with chronic constipation, assessed the effect of prunes on stool consistency only and were meta‐analysed [14]. Overall, prunes had no greater impact on stool consistency compared to psyllium supplements (MD: +0.45 points in the Bristol Stool Form Scale, 95% CI: −0.24, 1.14 points).

**Kiwifruits, prunes and symptoms**

26. **There is no difference between kiwifruits and psyllium on frequency of straining in constipation.**
*Level of evidence: Very low*
*Strength of recommendation: Qualified*
27. **There is no difference between prunes and psyllium on severity of straining in constipation.**
*Level of evidence: Very low*
*Strength of recommendation: Qualified*

Three RCTs, including 192 people with chronic constipation, investigated the effect of kiwifruits on the frequency of straining [14]. Overall, kiwifruits had no greater impact on frequency of straining compared to psyllium supplements (SMD: −1.62, 95% CI: −4.45, 1.21).

Two RCTs, including 89 people with chronic constipation, investigated the effect of prunes on severity of straining [14]. Overall, prunes had no greater impact on severity of straining compared to psyllium supplements (SMD: −0.13, 95% CI: −0.69, 0.43).

**Kiwifruits: Good Practice statements**

- Consumption of 2–3 kiwifruit daily for at least 4 weeks may be recommended in constipation (evidence‐based recommendation).
- Kiwifruit without skin may be recommended for constipation (evidence‐based recommendation). While not assessed in studies, keeping the skin on will provide additional fibre, which may potentially be beneficial, but may also potentially increase side effects (expert opinion recommendation).
- Kiwifruit may be a preferred option over psyllium in people with constipation who experience side effects such as bloating, abdominal pain and flatulence (evidence‐based recommendation).

### 5.2 Rye Bread

Statements on rye bread were derived from the evidence generated in a systematic review and meta‐analysis of 2 RCTs that included 48 people with chronic constipation and investigated the effect of rye bread (6 or 8 slices/day) compared to white bread for 3 weeks [14].

**Rye bread and stool output**

28. **Rye bread is more effective than white bread at increasing stool frequency in constipation.**
*Level of evidence: low*
*Strength of recommendation: qualified*

Two RCTs, including 48 people with chronic constipation, assessed the effect of rye bread on stool frequency compared to white bread. Overall, rye bread significantly increased stool frequency by +0.43 bowel movements/week (95% CI: 0.03, 0.83 bowel movements/week) compared to white bread. However, the magnitude of effect was small, which is reflected in the strength of recommendation (qualified). In addition, the dose was 6 or 8 slices of rye bread per day, which may not be a realistic or manageable dose for some patients.

**Rye bread and symptoms**

29. **Rye bread worsens global symptoms of constipation compared to white bread.**
*Level of evidence: low*
*Strength of recommendation: qualified*

Two RCTs, including 48 people with chronic constipation, assessed the effect of rye bread on global symptoms of constipation (including severity of abdominal pain, flatulence, borborygmi, abdominal bloating, constipation, diarrhoea) compared to white bread using a questionnaire with scores ranging from 0 to 18 (higher score, worse global symptoms). Overall, rye bread increased the global symptom score by +2.00 points (95% CI: 0.48, 3.53) compared to white bread. Therefore, rye bread worsened global symptoms. However, the magnitude of the effect was small and may not be clinically important to patients.

**Rye bread: Good Practice statement**

Consuming 6–8 slices of rye bread daily for at least 3 weeks may be recommended in constipation, (evidence‐based recommendation), however, this may not be realistic or manageable for some patients.

### 5.3 High Mineral‐Content Water

Statements on high mineral‐content water were derived from the evidence generated in a systematic review and meta‐analysis of 4 RCTs that included 755 people with chronic constipation and investigated the effect of high mineral‐content water, compared to low mineral‐content water [14].

**High mineral‐content water and response to treatment**

30. **High mineral‐content water is more effective than low mineral‐content water at increasing the number of people with constipation who have a clinical benefit.**
*Level of evidence: low*
*Strength of recommendation: strong*

Three RCTs (one of which had two test arms), including 539 people with chronic constipation, assessed the effect of high mineral‐content water on symptomatic response to treatment compared to low mineral water [14]. Overall, high mineral‐content water increased the likelihood of a beneficial response to treatment by 47% compared to low mineral‐content water (RR: 1.47, 95% CI: 1.20, 1.81). The doses provided daily were 0.5, 1 and 1.5 L per day for 2–6 weeks.

**High mineral‐content water and stool output**

31. **There is no difference between high mineral‐content water compared to low mineral‐content water on stool frequency in constipation.**
*Level of evidence: low*
*Strength of recommendation: strong*

Four RCTs (one of which had two test arms), including 638 people with chronic constipation, assessed the effect of high mineral‐content water on stool frequency compared to low mineral‐content water. Overall, high mineral‐content water had no impact on stool frequency, compared to low mineral‐content water (MD: +0.41 bowel movements/week, 95% CI: −0.05, 0.88 bowel movements/week).

**High mineral‐content water and symptoms**

32. **There is no difference between high mineral‐content water and low mineral‐content water on global symptoms of constipation.**
*Level of evidence: moderate*
*Strength of recommendation: strong*

Two RCTs, including 302 people with chronic constipation, assessed the effect of high mineral water on global symptom scores compared to low mineral water. Overall, high mineral‐content water had no impact on global symptoms of constipation compared to low mineral‐content water (SMD: −0.04, 95% CI: −0.27, 0.18).

**High mineral‐content water and adverse events**

33. **There is no difference between high mineral‐content water and low mineral‐content water on the severity of abdominal pain in constipation.**
*Level of evidence: moderate*
*Strength of recommendation: strong*

Three RCTs (one of which had two test arms), including 546 people with chronic constipation, assessed the effect of high mineral‐content water on the severity of abdominal pain compared to low mineral water. Overall, high mineral‐content water had no impact on the severity of abdominal pain compared to low mineral water (SMD: −0.17, 95% CI: −0.42, 0.08).

**High mineral‐content water and quality of life**

34. **There is no difference between high mineral‐content water and low mineral‐content water on global quality of life in constipation.**
*Level of evidence: low*
*Strength of recommendation: strong*

Two RCTs (one of which had two test arms), including 320 people with chronic constipation, assessed the effect of high mineral‐content water on global quality of life compared to low mineral‐content water. Overall, high mineral‐content water had no impact on global quality of life compared to low mineral‐content water (SMD: 0.13, 95% CI: −0.10, 0.36).

**High mineral‐content water: Good Practice statements**

Drinking 0.5–1.5 L/d high mineral‐content water for 2–6 weeks may improve symptomatic response to treatment, but not other symptoms (evidence‐based recommendation).

- There are no standardised thresholds defining the mineral content of high mineral‐content water for improving constipation. The table below shows the content of key minerals found in the high mineral‐content waters that were investigated in research studies, and thus those used to base the guidelines statements on, in comparison to the mineral water content of tap water in the United Kingdom (for reference). Patients should be encouraged to consume water with a mineral content comparable to that used in previous research studies (evidence‐based recommendation).

**Table:** 

Mineral (mg/L)	High mineral‐content water (used in research studies) [14]	UK tap water [21]
Calcium	370–573	5–128
Magnesium	105–1000	0.6–32
Sulphate	1530–2000	3–112
Sodium	28.9–1600	3–58

### 5.4 Whole Diet Interventions

Only one RCT investigated the effect of a high fibre diet on chronic constipation, and no RCTs were found on other whole diet approaches (e.g., Mediterranean diet) [14]. As a result, no recommendation statements were produced for any whole‐diet interventions in chronic constipation.

## 6 Research Recommendations

The Guideline Steering Committee developed a list of research recommendations based on the findings of the systematic reviews and the GRADE process in developing these recommendation statements. These are summarised in Box 1.

Box 1 **Research recommendations for dietary interventions in chronic constipation.**

1. *Probiotic supplements:* It is still unclear which strains of probiotics are effective in constipation. Further well‐designed RCTs examining the effect of appropriately characterised probiotic strains in chronic constipation are needed.
2. *Synbiotic supplements:* The evidence for synbiotic supplements in chronic constipation is sparse. Further RCTs examining different combinations of probiotic and prebiotic/fibre supplements in chronic constipation are needed.
3. *Prunes:* Evidence from a systematic review and meta‐analysis of RCTs suggests that prunes are not more effective than psyllium in improving constipation outcomes [14]. Psyllium is itself an effective treatment for chronic constipation. However, these studies did not follow a truly comparative evaluation design in that studies were not equivalence trials; hence, it is not possible to conclude that prunes are as effective as psyllium. Future studies are needed to establish the effectiveness of prunes in people with chronic constipation, either through a placebo‐controlled RCT or an appropriately designed equivalence RCT compared with psyllium or other effective interventions.
4. *Fermented foods:* There is increased interest in research into the use of fermented foods to improve chronic constipation. For example, a few small non‐randomised and uncontrolled trials on kefir have been undertaken, indicating potential beneficial effects on constipation [22, 23]. High‐quality RCTs are now required to establish the impact of fermented foods, such as kefir, in people with constipation.
5. *Food products with inactivated bacteria:* There is little research on the effect of food products with inactivated bacteria in chronic constipation. An RCT of pasteurised yoghurt with inactivated bacteria showed promising effects on the relief of constipation symptoms [24]. Future studies are needed to establish the effectiveness of food products with inactivated bacteria in people with constipation.
6. *High fibre foods and diets:* An increase in fibre intake is often recommended in the management of chronic constipation, alongside additional fluid intake [9, 25]. The vast majority of evidence is for fibre supplements, not high fibre foods or diets. There is promising evidence from single RCTs and uncontrolled trials of different high fibre foods in constipation (e.g., mango, figs, oat bran, high fibre cereal, and flaxseed flour [14]) that warrant adequately powered and appropriately controlled RCTs.
7. While some studies exist on high fibre foods in chronic constipation, there are currently no RCTs evaluating the effect of a high fibre diet from a range of foods in the diet in chronic constipation. Future RCTs are required to establish whether high fibre diets are effective in chronic constipation, and whether any beneficial effects might be enhanced with appropriate concurrent fluid intake.
8. *Additional fluids:* There is currently a lack of evidence to support that additional fluid consumption alone is beneficial in chronic constipation. Only one study of water supplementation (2 L/d) alongside a diet containing 25 g/d fibre has been performed and was effective in increasing stool frequency and decreasing laxative use in chronic constipation, compared to control [26]. Future RCTs are needed to establish whether increasing fluid intake beyond habitual levels is effective in chronic constipation.
9. *Caffeine:* No RCTs have been conducted on the effect of caffeine on chronic constipation, and future trials are needed to establish this.
10. *Senna:* Senna is a popular treatment in constipation, and its use has been recommended previously in guidelines [9]. Despite two placebo‐controlled RCTs individually demonstrating effectiveness of senna supplements on constipation outcomes, compared to placebo, when these were meta‐analysed together, the overall effect was no longer significant, albeit with significant heterogeneity [11]. The many remaining studies of senna include numerous uncontrolled trials. Future high‐quality placebo‐controlled RCTs are needed to establish the effectiveness of senna supplements in chronic constipation.
11. *Vitamin C:* Anecdotally, vitamin C has been hypothesised to improve symptoms of chronic constipation. This originates from the fact that a possible side effect of high doses of vitamin C is diarrhoea, and hence, it may have a laxative effect in people with constipation [27]. However, no RCTs investigating the effect of vitamin C in chronic constipation have been conducted. Thus, RCTs are needed to assess the impact of vitamin C in people with chronic constipation.
12. *Trigger foods:* Although several foods (e.g., rice, bananas) are widely perceived to worsen constipation symptoms [28], there is very limited research to support such claims. RCTs are needed to identify dietary components, food, diets and dietary behaviours that may trigger or worsen symptoms of constipation.

## 7 Discussion

These are the first evidence‐based guidelines for the dietary management of chronic constipation. Overall, 59 evidence‐based recommendation statements were produced and graded for their level of evidence and strength of recommendation. A clinician‐friendly guide was also developed based upon the recommendation statements to provide a practical tool that facilitates the adoption of the guidelines in clinical practice. In addition to the evidence‐based recommendation statements, 12 research recommendations were proposed to address unmet needs and gaps in the literature.

While previous medical guidelines for the management of constipation have included dietary recommendations, they almost exclusively focus on fibre quantity and/or type, omitting a variety of other effective dietary interventions that have been previously studied [9, 10]. For the first time, the current guidelines offer recommendations for various dietary interventions that have not been previously included in medical guidelines. For example, recommendations have been made for magnesium oxide supplements, kiwifruits, and high mineral‐content water, highlighting that these may improve cardinal symptoms of constipation.

To ensure these were strictly evidence‐based guidelines, recommendation statements were only produced for dietary strategies for which evidence from research studies exists, and recommendations for interventions for which evidence is lacking were not included. Previous medical guidelines have recommended that patients follow a high fibre diet. However, surprisingly, only one RCT was identified in our systematic review and meta‐analysis that assessed the effect of a high fibre diet (where fibre comes from a range of different foods/drinks across multiple food groups) in chronic constipation [14], and thus, no recommendations could be made for a high fibre diet. However, recommendation statements for several specific high‐fibre supplements and foods, for which evidence exists from multiple RCTs, have been made (e.g., psyllium supplements, kiwifruits, rye bread). Similarly, while senna supplements are widely recommended, they were found to be ineffective in constipation. While the individual RCTs separately showed a significant improvement in certain constipation outcomes, when they were meta‐analysed together, there was no longer a significant impact, and a significant heterogeneity was detected between the trials, potentially contributing to the loss of the initially observed effect. As a result, the recommendation statements highlight that senna supplements have no impact on the assessed constipation outcomes.

A rigorous approach was adopted to develop these dietary guidelines. First, they were developed by a multidisciplinary expert Guideline Steering Committee, consisting of expert dietitians and nutritionists, a general practitioner, a gastroenterologist and a gut physiologist, ensuring the guidelines meet the needs of patients who present in various clinical settings and types of clinical expertise. However, no patients, who are experts by experience, were included in the committee. Second, to identify and evaluate all previous evidence for any dietary treatment in chronic constipation, four rigorous systematic reviews and meta‐analyses of a total of 75 RCTs were conducted [11, 12, 13, 14]. These guidelines were then developed using the evidence generated by these meta‐analyses. Third, the GRADE approach was followed to assess the level of evidence and strength of recommendation, ensuring a meticulous approach to assessing the evidence and statements. Overall, of the 59 recommendation statements, 12 (20%) had a very low level of evidence, 39 (66%) had low, and 8 (14%) had a moderate level of evidence. Thirty‐two (54%) were a qualified recommendation, and 27 (46%) were a strong recommendation. The fact that most of the recommendation statements were qualified and of ‘very low’ or ‘low’ level of evidence highlights the current poor quality of research dietary studies in chronic constipation and the need to conduct high‐quality, fully‐powered dietary RCTs, using appropriate controls, standardised definitions and assessment tools/techniques. Lastly, the high cut‐off used (≥ 85%) for acceptable agreement among the Guideline Steering Committee emphasises the very strong consensus reached in support of the recommendation statements in these guidelines.

A common issue in the literature is the lack of a consistent definition of chronic constipation. While formal diagnostic criteria exist for chronic (primary) constipation, the Rome IV criteria, there is disagreement on perceptions of what constipation is between patients and clinicians, as well as between such perceptions and the formal criteria [29]. Multiple terminologies have been used to define chronic constipation in the literature, including primary constipation, idiopathic constipation, functional constipation, and evacuation disorders. The Guideline Steering Committee agreed it was important that these dietary guidelines address the needs of most patients who commonly present and seek constipation treatment in clinical practice. For this reason, a broader definition of constipation was adopted, including those diagnosed based on widely‐recognised clinical diagnostic criteria (e.g., the Rome criteria [17]), author‐defined or clinician‐defined criteria/diagnosis, participant‐defined criteria (e.g., self‐reported constipation) or the presence of at least one symptom indicative of constipation. Constipation secondary to medications or other conditions, as well as constipation‐related complications (e.g., faecal incontinence), were not covered in these guidelines; however, the recommendation statements could be applied in secondary constipation with caution and as clinically applicable.

Instead of the recommendation statements addressing the effect of dietary interventions in ‘constipation’ in general, each statement referred to specific constipation outcomes, including response to treatment, stool frequency and consistency, global and specific gut symptoms, adverse events, and global and specific QoL. This was in recognition of the highly diverse symptoms patients with constipation experience and the varied definitions clinicians and patients use to characterise constipation [29, 30]. In addition, while certain dietary interventions may improve one cardinal aspect of constipation (e.g., stool frequency), they may have no impact on another (e.g., straining), or they may even worsen another (e.g., flatulence). Therefore, an important strength of these guidelines is that by having outcome‐specific recommendation statements, clinicians can provide personalised dietary advice tailored to the patients' needs, symptoms and concerns.

These guidelines provide positive recommendation statements, where a dietary intervention improved a constipation outcome (e.g., ‘psyllium supplements reduce the severity of straining in constipation’), but also null statements, where a dietary intervention had no impact on a constipation outcome (e.g., ‘*Lactobacillus casei* Shirota supplements do not impact global symptoms of constipation’), and negative statements, where a dietary intervention worsened a constipation outcome (e.g., ‘Rye bread worsens global symptoms of constipation compared to white bread’).

Overall, 27/59 (46%) recommendations statements stated an improvement in the outcome of interest, 5/59 (8%) also stated a null effect compared to a positive control (i.e., psyllium supplement), 37/59 (63%) stated a null effect compared to a negative control (e.g., placebo), and 2/59 (3%) stated a worsening effect in the outcome of interest. By incorporating not only positive statements, but also null and negative ones, the guidelines allow clinicians to remain best informed on the state of the available evidence on supplements and foods that are commonly used by patients and potentially perceived as being effective. This enables clinicians to engage patients in evidence‐based discussions, addressing their current dietary practices and potential misconceptions effectively.

Clinicians should also consider individual co‐morbidities or dietary restrictions, as some interventions, such as high mineral‐content water or magnesium supplements, may be unsuitable for people with dietary restrictions (e.g., low magnesium or sodium diet). In addition, high mineral content water may contain high concentrations of sodium, and in those who already have a high intake of sodium, it may contribute to intakes that exceed national recommendations for salt intake, with its associated impact on health.

The systematic reviews of the literature on the effect of diet on constipation revealed a major unmet need and areas in which future research is warranted. As a result, the Guideline Steering Committee developed 12 research recommendations on supplements, foods, drinks and whole diets for which high‐quality research is needed to improve the evidence base in constipation and, consequently, clinical care. It is noteworthy that most RCTs identified in the process of developing the guidelines studied supplements, a smaller number studied foods and drinks, and even fewer studied whole diets. A number of studies were industry‐funded, and it is possible this may have driven research directions and, thus, increased the availability of research on specific dietary interventions. Moving forward, it is important that research studies use interventions and outcomes that are based on scientific and mechanistic relevance, as well as importance to patients.

Finally, it is worth noting that these guidelines were based on evidence from studies published by July 2023 and, therefore, newer studies published thereafter were not included in the development of these guidelines.

In conclusion, these BDA guidelines are the first ever dietary guidelines for the management of chronic constipation, providing recommendations on various dietary interventions and for several constipation‐related outcomes. A practical tool has also been developed to facilitate their adoption in clinical practice. Overall, specific fibre supplements (i.e., psyllium supplements), certain probiotic strains, magnesium oxide supplements, kiwifruits, rye bread and high mineral water have all been shown to improve specific constipation outcomes. However, most of the findings originate from a low level of evidence, and further research is warranted to strengthen and improve clinical care.

## Author Contributions

Eirini Dimidi conceived the project idea. Eirini Dimidi and Kevin Whelan developed the overall project design; Eirini Dimidi, Alice van der Schoot, Kevin Barrett, Adam D. Farmer, Miranda C. Lomer, S. Mark Scott and Kevin Whelan refined the guidelines remit and methodology; Alice van der Schoot drafted the initial recommendation statements; Eirini Dimidi, Alice van der Schoot, Kevin Barrett, Adam D. Farmer, Miranda C. Lomer, S. Mark Scott and Kevin Whelan voted on the recommendation statements; Eirini Dimidi drafted the manuscript; all authors reviewed and approved the final manuscript.

## Conflicts of Interest

Eirini Dimidi has received an education grant from Alpro, research grants from the Almond Board of California, the International Nut and Dried Fruit Council and Nestec Ltd., and has served as a consultant for Puratos and Danone. Alice van der Schoot is currently funded by a grant from the Almond Board of California. Kevin Barrett has received speaker fees and honoraria for writing articles for Bimuno and Symprove. Miranda C. Lomer has received speaker fees from Janssen, Mayoly and AbbVie. Miranda C. Lomer is a Course Director of low FODMAP courses for dietitians. Miranda C. Lomer receives royalties from Wiley Publishing in relation to an academic textbook on nutrition and dietetics in gastroenterology. Kevin Whelan has received research grants related to diet and gut health and disease from the Almond Board of California, Danone, and the International Nut and Dried Fruit Council and has received speaker fees from Danone and Yakult. Kevin Whelan is the holder of a joint patent to use volatile organic compounds as biomarkers in irritable bowel syndrome (PCT/GB2020/051604), for which there is currently no product on the market. Kevin Whelan receives royalties from Wiley Publishing in relation to an academic textbook on nutrition and dietetics in gastroenterology. The other authors declare no conflicts of interest.

## 1 Peer Review

The peer review history for this article is available at https://www.webofscience.com/api/gateway/wos/peer-review/10.1111/jhn.70133.

## Supporting information


**Table S1:** Detailed search strategies for studies investigating the effect of dietary interventions on chronic constipation in adults. **Table S2:** GRADE critical outcomes.

## Data Availability

The data that support the findings of this study are available from the corresponding author upon reasonable request.
