# Supplementary material for: British Dietetic Association Guidelines for the Dietary Management of Chronic Constipation in Adults
Source: J Hum Nutr Diet. 2025 Oct 13;38(5):e70133. doi: 10.1111/jhn.70133 (PMC12517116; doi:10.1111/jhn.70133)
Supplement: Supplementary file 1 — Table S1: Detailed search strategies for studies investigating the effect of dietary interventions on chronic constipation in adults. Table S2: GRADE critical outcomes. [file JHN-38-0-s001.docx]

**Table S1:** Detailed search strategies for studies investigating the effect of dietary interventions on chronic constipation in adults.

| Systematic review (search date) | MEDLINE | EMBASE | CENTRAL | WEB OF SCIENCE |
| --- | --- | --- | --- | --- |
| **Fibre supplements(18th**  **March 2022)** | Constipation.mp. OR exp Constipation/ OR “functional constipation”.mp. OR “primary constipation”.mp. OR “chronic constipation”.mp. OR “idiopathic constipation”.mp. OR “slow transit constipation”.mp. OR constipated.mp. OR “def?ecation disorder*”.mp. OR “Evacuation disorder*”.mp. OR “gastrointestinal transit”.mp. OR exp Gastrointestinal Transit/ OR “gut transit”.mp. OR “slow transit”.mp. OR “hard stool*”.mp. OR “lumpy stool*”.mp. OR “hard f?eces”.mp. OR straining.mp. OR “incomplete evacuation”.mp. OR infrequent bowel movement*.mp.  **AND**  exp Adult/ or exp Young Adult/ OR adult*.mp. OR elderly.mp. OR exp Aged/ OR individual*.ab. OR volunteer*.ab. OR participant*.ab. OR subject*.ab. OR patient*.ab. OR human*.ab.  **AND**  Dietary fiber”.mp. OR exp Dietary Fiber/ OR “Dietary fibre”.mp. OR Fiber.mp. OR Fibre.mp. OR exp Plant Extracts/ OR plant extract*”.mp. OR “resistant starch”.mp. OR “resistant maltodextrin”.mp. OR “digestion-resistant maltodextrin”.mp. OR isomaltodextrin.mp. OR cellulose.mp. OR exp Cellulose/ OR pectin.mp. or exp Pectins/ OR lignin.mp. OR exp Lignin/ OR exp beta-Glucans/ ORbeta-glucan*.mp. OR “soluble maize fib*”.mp. OR “Soluble corn fib*”.mp. OR pullulan.mp. OR glucomannan.mp. OR konjac.mp. OR exp Amorphophallus/ OR Galactomannan.mp. OR Arabinan*.mp. OR Arabinogalactan*.mp. OR Arabinoxylan*.mp. OR Polydextrose.mp. OR bran.mp. cereal.mp. OR “acacia gum”.mp. or exp Gum Arabic/ OR “Partially hydroly?ed guar gum”.mp. OR “guar gum”.mp. OR psyllium.mp. OR exp Psyllium/ OR “Ispaghula husk”.mp. OR “Plantago ovata”.mp. OR exp Plantago/ OR metamucil.mp. OR Fybogel.mp. OR exp Fructans/ or fructan*.mp. OR inulin.mp. OR exp Inulin/ OR “inulin type fruct*”.mp. OR chicory.mp. OR exp Chicory/ OR exp Oligosaccharides/ OR oligosaccharide*.mp. OR fructooligosaccharide*.mp.  OR oligofructose.mp. OR galactooligosaccharide*.mp. OR transgalactooligosaccharide*.mp. OR isomaltooligosaccharide*.mp OR exp Galactans/ or galactan*. mp.OR mannanoligosaccharide*.mp. OR xylooligosaccharide*.mp. OR oligomer*.mp. OR sc-FOS.mp. OR methylcellulose.mp. OR exp Methylcellulose/  **AND**  Randomi?ed controlled trial.pt. OR controlled clinical trial.pt. OR exp Controlled Clinical Trial/ OR exp Clinical Trial/ OR randomi?ed.ti,ab. OR randomly.ti,ab. OR random*.ti,ab OR placebo.ti,ab. OR trial.ti,ab. OR control*.ti,ab OR “clinical trial”.ti,ab OR clinical trials as topic.sh. **NOT** exp animals/ not humans.sh. OR (rat or rats or mouse or mice or swine or porcine or murine or sheep or lambs or pigs or piglets or rabbit or rabbits or cat or cats or dog or dogs or cattle or bovine or monkey or monkeys or trout or marmoset*).ti.  *1155 records* | exp constipation/ OR exp chronic constipation/ OR constipation.mp. OR ‘functional constipation’.mp. OR ‘primary constipation’.mp. OR ‘chronic constipation’.mp. OR ‘idiopathic constipation’.mp. OR ‘slow transit constipation’.mp. OR constipated.mp. OR exp defecation disorder/ OR ‘Def?ecation disorder*’.mp. OR ‘evacuation disorder*’.mp. OR ‘Gastrointestinal transit’.mp. OR exp gastrointestinal transit/ OR ‘gut transit’.mp. OR ‘slow transit’.mp. OR ‘hard stool*'.mp. OR ‘lumpy stool*’.mp. OR ‘hard f?eces’.mp. OR exp hard feces/ OR straining.mp. OR ‘incomplete evacuation’.mp. OR ‘infrequent bowel movement*’.mp.  **AND**  exp young adult/ OR exp adult/ OR adult*.mp. OR elderly.mp. OR exp aged/ OR individual*.ab. OR volunteer*.ab. OR participant*.ab. OR subject*.ab. OR patient*.ab. OR human*.ab.  **AND**  ‘dietary fiber’.mp. OR exp dietary fiber/ OR ‘dietary fibre’.mp. OR fiber.mp. OR exp fiber/ OR fibre.mp. OR prebiotic*.mp. OR ‘non starch polysaccharide*’.mp.OR exp plant extract/ OR ‘plant extract*’.mp. OR ‘resistant starch’.mp. OR ‘resistant maltodextrin’.mp. OR ‘digestion-resistant maltodextrin’.mp. OR isomaltodextrin.mp. OR exp cellulose/ OR cellulose.mp. OR exp pectin/ OR pectin.mp. OR exp lignin/ OR lignin.mp. OR exp beta glucan/ OR beta-glucan*.mp. OR ‘soluble maize fib*’.mp.  OR ‘soluble corn fib*’.mp. OR pullulan.mp. OR exp pullulan/ OR glucomannan.mp. OR konjac.mp. OR exp Amorphophallus konjac/ OR Galactomannan.mp. OR exp galactomannan/ OR arabinan*.mp. OR exp arabinogalactan/ OR arabinogalactan*.mp . OR exp arabinoxylan/ or arabinoxylan*.mp. OR polydextrose.mp. OR exp bran/ OR bran.mp. OR cereal.mp. OR exp cereal/ OR 'acacia gum’.mp. OR exp gum arabic / OR ‘partially hydroly?ed guar gum’.mp. OR ‘guar gum’.mp. OR exp guar gum/ OR psyllium.mp. OR exp ispagula/ OR ‘ispaghula husk’.mp. OR ‘plantago ovata’.mp. OR exp Plantago ovata/ OR metamucil.mp. OR fybogel.mp. OR exp fructan/ OR fructan*.mp. OR exp inulin/ or inulin.mp. OR ‘inulin type fruct*’.mp. OR chicory.mp. OR exp chicory/ OR exp oligosaccharide/ OR oligosaccharide*.mp OR exp fructose oligosaccharide/ or fructooligosaccharide*.mp. OR oligofructose.mp. OR exp galactose oligosaccharide/ or galactooligosaccharide*.mp. OR transgalactooligosaccharide*.mp. OR isomaltooligosaccharide*.mp. OR exp galactan/ or galactan*.mp. OR mannanoligosaccharide*.mp. OR xylooligosaccharide*.mp. OR exp oligomer/ OR oligomer*.mp. OR sc-FOS.mp. OR methylcellulose.mp. OR exp methylcellulose  **AND**  exp randomized controlled trial/ OR exp controlled clinical trial/ OR random$.ti,ab. OR exp randomization/ OR exp intermethod comparison/ OR placebo.ti,ab. OR (compare or compared or comparison).ti. OR ((evaluated or evaluate or evaluating or assessed or assess) and (compare or compared or comparing or comparison)).ab. OR (open adj label).ti,ab. OR ((double or single or doubly or singly) adj (blind or blinded or blindly)).ti,ab. OR exp double blind procedure/ OR parallel group$1.ti,ab. OR (crossover or cross over).ti,ab. OR ((assign$ or match or matched or allocation) adj5 (alternate or group$1 or intervention$1 or patient$1 or subject$1 or participant$1)).ti,ab. OR (assigned or allocated).ti,ab. OR (controlled adj7 (study or design or trial)).ti,ab. OR (volunteer or volunteers).ti,ab. OR exp human experiment/ OR trial.ti. NOT (random$ adj sampl$ adj7 ('cross section$' or questionnaire$1 or survey$ or  database$1)).ti,ab. not (comparative study/ or controlled study/ or randomi?ed controlled.ti,ab. or randomly assigned.ti,ab.) OR Cross-sectional study/ not (randomized controlled trial/ or controlled clinical study/ or controlled study/ or randomi?ed controlled.ti,ab. or control group$1.ti,ab.) OR (((case adj control$) and random$) not randomi?ed controlled).ti,ab. **OR** (Systematic review not (trial or study)).ti. OR (nonrandom$ not random$).ti,ab. OR 'Random field$'.ti,ab. OR (random cluster adj3 sampl$).ti,ab. OR (review.ab. and review.pt.) not trial.ti. OR 'we searched'.ab. and (review.ti. or review.pt.) OR 'update review'.ab. OR (databases adj4 searched).ab. OR (rat or rats or mouse or mice or swine or porcine or murine or sheep or lambs or pigs or piglets or rabbit or rabbits or cat or cats or dog or dogs or cattle or bovine or monkey or monkeys or trout or marmoset$1).ti. and animal experiment/ OR Animal experiment/ not (human experiment/ or human/)  *3106 records* | Title, abstract, keywords:  Dietary Fiber OR Fibre OR Prebiotic OR Psyllium  **AND**  Search all text:  Constipation  *667 records* | constipation OR “functional constipation” OR “primary constipation” OR “chronic constipation” OR “idiopathic constipation” OR “slow transit constipation” OR constipated OR “def?ecation disorder*” OR “Evacuation disorder*” OR “gastrointestinal transit” OR “gut transit” OR “slow transit” OR “hard stool*” OR “lumpy stool*” OR “hard f?eces” OR straining OR “incomplete evacuation” OR “infrequent bowel movement*”) as TOPIC  **AND**  Adult* OR elderly OR individual* OR volunteer* OR participant* OR subject* OR patient* OR human*) as TOPIC  **AND**  (Fiber OR Fibre OR “Dietary fiber” OR “Dietary fibre” OR prebiotic* OR “non starch polysaccharide*” OR “plant extract*” OR “resistant starch” OR “resistant maltodextrin” OR “digestion-resistant maltodextrin” OR isomaltodextrin OR cellulose OR pectin OR lignin OR “beta-glucan*” OR “soluble maize fib*” OR “Soluble corn fib*” OR pullulan OR glucomannan OR konjac OR  Galactomannan OR Arabinan* OR Arabinogalactan* OR Arabinoxylan* OR Polydextrose OR bran OR cereal OR “acacia gum” OR “Partially hydroly?ed guar gum” OR “guar gum” OR psyllium OR “Ispaghula husk” OR “Plantago ovata” OR metamucil OR Fybogel OR fructan* OR inulin OR “inulin type fruct*” OR chicory OR oligosaccharide* OR fructooligosaccharide* OR oligofructose OR galactooligosaccharide* OR transgalactooligosaccharide* OR isomaltooligosaccharide* OR galactan* OR mannanoligosaccharide* OR xylooligosaccharide* OR oligomer* OR "sc-FOS" OR methylcellulose) as  TOPIC  **AND**  “Randomi?ed controlled trial” OR “controlled clinical trial” OR “Clinical Trial” OR randomi?ed OR randomly OR random* OR placebo OR trial OR control*) as TOPIC  *3906 records* |
| **Probiotics and synbiotics (10^th^ July 2022)** | constipation.mp. or exp Constipation/ OR “functional constipation”.mp. OR “primary constipation”.mp. OR “chronic constipation”.mp. OR “idiopathic constipation”.mp. OR “slow transit constipation”.mp. OR constipated.mp.  OR “def?ecation disorder*”.mp. OR “Evacuation disorder*”.mp. OR “gastrointestinal transit”.mp. or exp Gastrointestinal Transit/ OR “gut transit”.mp. OR “slow transit”.mp. OR “hard stool*”.mp. OR “lumpy stool*”.mp. OR “hard f?eces”.mp. OR straining.mp. OR “incomplete evacuation”.mp. OR “infrequent bowel movement*”.mp.  **AND**  exp Adult/ or exp Young Adult/ OR adult*.mp. OR elderly.mp. or exp Aged/ OR individual*.ab.  OR volunteer*.ab. OR participant*.ab. OR subject*.ab. OR patient*.ab. OR human*.ab.  **AND**  Bacteria/ OF Probiotics/ OR exp Synbiotics/ OR synbiotic*.mp OR symbiotic*.mp OR exp Dietary Fiber/ OR exp Prebiotics/ OR Escherichia coli/ OR Lactobacillus/ OR probiotic*.mp. OR Bifidobacterium/ OR bifidobacterium.mp. OR lactobacillus.mp. OR “Lactobacillus acidophilus”.mp. OR exp Lactobacillus acidophilus/ OR “lactobacillus casei”.mp. OR Lactobacillus Casei/ OR “lactobacillus rhamnosus”.mp. OR Lactobacillus rhamnosus/ OR “bifidobacterium lactis”.mp. OR “bifidobacterium animalis”.mp. OR exp Bifidobacterium animalis/ OR bifidobacteria.mp. OR Saccharomyces/ OR “saccharomyces boulardii”.mp. OR “Escherichia coli nissle”.mp. OR “escherichia coli” OR exp Bacillus/ OR Bacillus.mp OR exp Bacillus coagulans/OR exp Bacillus subtilis/ OR “microbial cell preparation*”.mp OR “fermented milk*”.mp.  **AND**  Randomi?ed controlled trial.pt. OR controlled clinical trial.pt. OR exp Controlled Clinical Trial/    OR exp Clinical Trial/ OR randomi?ed.ti,ab. OR randomly.ti,ab OR random*.ti,ab OR placebo.ti,ab. OR trial.ti,ab OR control*.ti,ab OR “clinical trial”.ti,ab OR clinical trials as topic.sh.  **NOT** exp animals/ not humans.sh. OR (rat or rats or mouse or mice or swine or porcine or murine or sheep or lambs or pigs or piglets or rabbit or rabbits or cat or cats or dog or dogs or cattle or bovine or monkey or monkeys or trout or marmoset*).ti.  *819 records* | exp constipation/ OR exp chronic constipation/ OR constipation.mp. ‘functional constipation’.mp. OR ‘primary constipation’.mp. OR ‘chronic constipation’.mp. OR ‘idiopathic constipation’.mp. OR ‘slow transit constipation’.mp. OR constipated.mp. OR exp defecation disorder/ or ‘Def?ecation disorder*’.mp. OR ‘evacuation disorder*’.mp. OR ‘Gastrointestinal transit’.mp. or exp gastrointestinal transit/ OR ‘gut transit’.mp.  OR ‘slow transit’.mp. OR ‘hard stool*'.mp. OR ‘lumpy stool*’.mp. OR ‘hard f?eces’.mp. or exp hard feces/ OR straining.mp. OR ‘incomplete evacuation’.mp. OR ‘infrequent bowel movement*’.mp.  **AND**  exp young adult/ OR exp adult/ OR adult*.mp. OR elderly.mp. or exp aged/ OR individual*.ab.  OR volunteer*.ab. OR participant*.ab. OR subject*.ab. OR patient*.ab. OR human*.ab.  **AND**  Exp Bacterium/ OR exp Probiotic agent/ OR exp Synbiotic agent/ OR symbiotic*.mp OR symbiotic*.mp OR exp Dietary fiber OR exp prebiotic agent/ OR Escherichia coli/ OR ‘escherichia coli’.mp OR Lactobacillus/ OR probiotic*.mp. OR Bifidobacterium/ OR bifidobacterium.mp. OR lactobacillus.mp. OR Lactobacillus acidophilus/ OR ‘Lactobacillus acidophilus’.mp. OR ‘lactobacillus casei’.mp. OR Lactobacillus casei/ OR ‘lactobacillus rhamnosus’.mp. OR Lactobacillus rhamnosus/ OR ‘bifidobacterium lactis’.mp. OR exp Bifidobacterium animalis/ OR ‘bifidobacterium animalis’.mp.OR bifidobacteria.mp. OR Saccharomyces/ OR ‘saccharomyces boulardii’.mp. OR Escherichia coli nissle.mp. OR exp Bacillus coagulans/ OR exp Bacillus/ OR Bacillus.mp OR exp Bacillus subtilis/ OR ‘microbial cell preparation*’.mp. OR ‘fermented milk*’.mp.  **AND**  exp randomized controlled trial/ OR exp controlled clinical trial/ OR random$.ti,ab. OR exp randomization/ OR exp intermethod comparison/ OR placebo.ti,ab. OR (compare or compared or comparison).ti. OR ((evaluated or evaluate or evaluating or assessed or assess) and (compare or compared or comparing or comparison)).ab. OR (open adj label).ti,ab. OR ((double or single or doubly or singly) adj (blind or blinded or blindly)).ti,ab. OR exp double blind procedure/ OR parallel group$1.ti,ab.  OR  (crossover or cross over).ti,ab. OR ((assign$ or match or matched or allocation) adj5 (alternate or group$1 or intervention$1 or patient$1 or subject$1 or participant$1)).ti,ab. OR (assigned or allocated).ti,ab. OR (controlled adj7 (study or design or trial)).ti,ab. OR (volunteer or volunteers).ti,ab. OR exp human experiment/ OR trial.ti.  **NOT**     (random$ adj sampl$ adj7 ('cross section$' or questionnaire$1 or survey$ or database$1)).ti,ab. not (comparative study/ or controlled study/ or randomi?ed controlled.ti,ab. or randomly assigned.ti,ab.) OR Cross-sectional study/ not (randomized controlled trial/ or controlled clinical study/ or controlled study/ or randomi?ed controlled.ti,ab. or control group$1.ti,ab.) OR (((case adj control$) and random$) not randomi?ed controlled).ti,ab.   OR (Systematic review not (trial or study)).ti. OR (nonrandom$ not random$).ti,ab. OR 'Random field$'.ti,ab. OR (random cluster adj3 sampl$).ti,ab. OR (review.ab. and review.pt.) not trial.ti. OR 'we searched'.ab. and (review.ti. or review.pt.) OR 'update review'.ab. OR (databases adj4 searched).ab. OR  (rat or rats or mouse or mice or swine or porcine or murine or sheep or lambs or pigs or piglets or rabbit or rabbits or cat or cats or dog or dogs or cattle or bovine or monkey or monkeys or trout or marmoset$1).ti. and animal experiment/ OR Animal experiment/ not (human experiment/ or human/)  *1702 records* | Title, abstract, keywords:  Probiotic OR synbiotic OR symbiotic  **AND**  Search all text:  Constipation  *542 records* | - |
| **Food, vitamin or mineral supplements (18^th^ February 2022)** | constipation.mp. or exp Constipation/ OR “functional constipation”.mp. OR “primary constipation”.mp. OR “chronic constipation”.mp. OR “idiopathic constipation”.mp. OR “slow transit constipation”.mp. OR constipated.mp.  OR “def?ecation disorder*”.mp. OR “Evacuation disorder*”.mp. OR “gastrointestinal transit”.mp. or exp Gastrointestinal Transit/ OR “gut transit”.mp. OR “slow transit”.mp. OR “hard stool*”.mp. OR “lumpy stool*”.mp. OR “hard f?eces”.mp. OR straining.mp. OR “incomplete evacuation”.mp. OR “infrequent bowel movement*”.mp.  **AND**  exp Adult/ or exp Young Adult/ OR adult*.mp. OR elderly.mp. or exp Aged/ OR individual*.ab.  OR volunteer*.ab. OR participant*.ab. OR subject*.ab. OR patient*.ab. OR human*.ab.  **AND**  Extract*.mp OR exp Plant Extracts/  OR exp Dietary Supplements/ OR food supplement*.mp. OR nutraceutical*.mp OR “fruit powder*”.mp OR “fruit capsule*”.mp OR “vegetable powder*”.mp OR “vegetable capsule*”.mp, OR “fruit supplement*”.mp OR “vegetable supplement*”.mp OR vitamin*.mp OR mineral*.mp  OR “kiwifruit capsule*”.mp OR exp Magnesium/ OR magnesium.mp OR senna.mp  **AND**  Randomi?ed controlled trial.pt. OR controlled clinical trial.pt. OR exp Controlled Clinical Trial/     OR exp Clinical Trial/ OR randomi?ed.ti,ab. OR randomly.ti,ab OR random*.ti,ab OR placebo.ti,ab. OR trial.ti,ab OR control*.ti,ab OR “clinical trial”.ti,ab OR clinical trials as topic.sh.   **NOT** exp animals/ not humans.sh. OR (rat or rats or mouse or mice or swine or porcine or murine or sheep or lambs or pigs or piglets or rabbit or rabbits or cat or cats or dog or dogs or cattle or bovine or monkey or monkeys or trout or marmoset*).ti.  *1111 records* | exp constipation/ OR exp chronic constipation/ OR constipation.mp. ‘functional constipation’.mp. OR ‘primary constipation’.mp. OR ‘chronic constipation’.mp. OR ‘idiopathic constipation’.mp. OR ‘slow transit constipation’.mp. OR constipated.mp. OR exp defecation disorder/ or ‘Def?ecation disorder*’.mp. OR ‘evacuation disorder*’.mp. OR ‘Gastrointestinal transit’.mp. or exp gastrointestinal transit/ OR ‘gut transit’.mp.  OR ‘slow transit’.mp. OR ‘hard stool*'.mp. OR ‘lumpy stool*’.mp. OR ‘hard f?eces’.mp. or exp hard feces/ OR straining.mp. OR ‘incomplete evacuation’.mp. OR ‘infrequent bowel movement*’.mp.  **AND**  exp young adult/ OR exp adult/ OR adult*.mp. OR elderly.mp. or exp aged/ OR individual*.ab.  OR volunteer*.ab. OR participant*.ab. OR subject*.ab. OR patient*.ab. OR human*.ab.  **AND**  Extract*.mp OR  exp plant extract/ OR exp dietary supplement/ OR “food supplement*”.mp OR nutraceutical*.mp OR “fruit powder*".mp. OR "fruit capsule*".mp. OR "vegetable powder*".mp. OR "vegetable capsule*".mp. OR "fruit supplement*".mp. OR "vegetable supplement*".mp. OR vitamin*.mp. OR exp vitamin/ OR mineral*.mp. OR exp mineral/ OR "kiwifruit capsule*".mp. OR exp magnesium/ OR magnesium.mp OR senna.mp  **AND**  exp randomized controlled trial/ OR exp controlled clinical trial/ OR random$.ti,ab. OR exp randomization/ OR exp intermethod comparison/ OR placebo.ti,ab. OR (compare or compared or comparison).ti. OR ((evaluated or evaluate or evaluating or assessed or assess) and (compare or compared or comparing or comparison)).ab. OR (open adj label).ti,ab. OR ((double or single or doubly or singly) adj (blind or blinded or blindly)).ti,ab. OR exp double blind procedure/ OR parallel group$1.ti,ab.  OR  (crossover or cross over).ti,ab. OR ((assign$ or match or matched or allocation) adj5 (alternate or group$1 or intervention$1 or patient$1 or subject$1 or participant$1)).ti,ab. OR (assigned or allocated).ti,ab. OR (controlled adj7 (study or design or trial)).ti,ab. OR (volunteer or volunteers).ti,ab. OR exp human experiment/ OR trial.ti.  **NOT** (random$ adj sampl$ adj7 ('cross section$' or questionnaire$1 or survey$ or database$1)).ti,ab. not (comparative study/ or controlled study/ or randomi?ed controlled.ti,ab. or randomly assigned.ti,ab.) OR Cross-sectional study/ not (randomized controlled trial/ or controlled clinical study/ or controlled study/ or randomi?ed controlled.ti,ab. or control group$1.ti,ab.) OR (((case adj control$) and random$) not randomi?ed controlled).ti,ab.   OR (Systematic review not (trial or study)).ti. OR (nonrandom$ not random$).ti,ab. OR 'Random field$'.ti,ab. OR (random cluster adj3 sampl$).ti,ab. OR (review.ab. and review.pt.) not trial.ti. OR 'we searched'.ab. and (review.ti. or review.pt.) OR 'update review'.ab. OR (databases adj4 searched).ab. OR  (rat or rats or mouse or mice or swine or porcine or murine or sheep or lambs or pigs or piglets or rabbit or rabbits or cat or cats or dog or dogs or cattle or bovine or monkey or monkeys or trout or marmoset$1).ti. and animal experiment/ OR Animal experiment/ not (human experiment/ or human/)  *2969 records* | Title, abstract, keywords:  Extract OR Vitamin OR Mineral OR Magnesium OR Vitamin c  **AND**  Search all text:  Constipation  *803 records* | - |
| **Foods, drinks, herbs, spices and whole diets (12^th^ July 2023)** | constipation.mp. or exp Constipation/ OR “functional constipation”.mp. OR “primary constipation”.mp. OR “chronic constipation”.mp. OR “idiopathic constipation”.mp. OR “slow transit constipation”.mp. OR constipated.mp. OR “def?ecation disorder*”.mp. OR “Evacuation disorder*”.mp. OR “gastrointestinal transit”.mp. or exp Gastrointestinal Transit/ OR “gut transit”.mp. OR “slow transit”.mp. OR “hard stool*”.mp. OR “lumpy stool*”.mp. OR “hard f?eces”.mp. OR straining.mp. OR “incomplete evacuation”.mp. OR infrequent bowel movement*.mp.  **AND**  exp Adult/ or exp Young Adult/ OR adult*.mp. OR elderly.mp. or exp Aged/ OR individual*.ab. OR volunteer*.ab. OR participant*.ab. OR subject*.ab. OR patient*.ab. OR human*.ab.  **AND**  food*.mp. OR exp food/ OR drink*.mp. OR beverage*.mp. OR exp beverage/ OR juice.mp. OR water.mp. OR alcohol*.mp. OR herb.mp. OR spice*.mp. OR exp spice/ OR diet*.mp. OR exp diet/ OR vegetarian.mp. OR vegan.mp. OR “plant based diet”.mp. OR pescatarian.mp. OR mediterranean.mp. OR fat.mp OR fibre.mp. OR fiber.mp. OR gluten.mp. OR FODMAP*.mp. OR fruit*.mp. OR exp fruit/ OR vegetable*.mp. OR exp vegetable/ OR legume*.mp. OR exp legumes/ OR nut*.mp. OR exp nut/ OR seed*.mp. OR exp seeds/ OR cereal*.mp. OR exp cereal/ OR grain*.mp. OR fermented.mp. OR dairy.mp. OR “non dairy”.mp. OR prune*.mp. OR kiwi*.mp. OR kefir.mp. OR tea.mp. OR coffee.mp. **NOT**  Isolate*.ti,ab. OR extract*.ti,ab.  **AND**  Randomi?ed controlled trial.pt. OR controlled clinical trial.pt. OR exp Controlled Clinical Trial/ OR exp Clinical Trial/ OR randomi?ed.ti,ab. OR randomly.ti,ab OR random*.ti,ab OR placebo.ti,ab. OR trial.ti,ab OR control*.ti,ab OR “clinical trial”.ti,ab OR clinical trials as topic.sh. OR exp Prospective Studies/ OR "single arm".ti,ab. OR "intervention study".ti,ab. OR exp Comparative Study/  **NOT**  exp animals/ not humans.sh. OR (rat or rats or mouse or mice or swine or porcine or murine or sheep or lambs or pigs or piglets or rabbit or rabbits or cat or cats or dog or dogs or cattle or bovine or monkey or monkeys or trout or marmoset*).ti. OR “in vitro”.ti.  *3112 records* | exp constipation/ OR exp chronic constipation/ OR constipation.mp. ‘functional constipation’.mp. OR ‘primary constipation’.mp. OR ‘chronic constipation’.mp. OR ‘idiopathic constipation’.mp. OR ‘slow transit constipation’.mp. OR constipated.mp. OR exp defecation disorder/ or ‘Def?ecation disorder*’.mp. OR ‘evacuation disorder*’.mp. OR ‘Gastrointestinal transit’.mp. or exp gastrointestinal transit/ OR ‘gut transit’.mp. OR ‘slow transit’.mp. OR ‘hard stool*'.mp. OR ‘lumpy stool*’.mp. OR ‘hard f?eces’.mp. or exp hard feces/ OR straining.mp. OR ‘incomplete evacuation’.mp. OR ‘infrequent bowel movement*’.mp  **AND**  exp young adult/ OR exp adult/ OR adult*.mp. OR elderly.mp. or exp aged/ OR individual*.ab. OR volunteer*.ab. OR participant*.ab. OR subject*.ab. OR patient*.ab. OR human*.ab.  **AND**  food.mp. OR exp food/ OR drink$.mp. OR beverage$.mp. OR exp beverage/ OR juice$.mp. OR water.mp. OR alcoholic.mp. OR herb$.mp. OR exp herb/ OR spice$.mp. er exp spice/ OR diet.mp. OR exp diet/ OR vegetarian.mp. OR vegan.mp. OR ‘plant based diet’.mp.. OR pescatarian.mp. OR mediterranean.mp. OR fat.mp. OR fiber.mp. OR fibre.mp OR gluten.mp. OR FODMAP$.mp. OR fruit$.mp. OR exp fruit/ OR vegetable$.mp. OR exp vegetable/ OR legume$.mp. OR exp legumes/ OR nut$.mp. OR exp nut/ OR seed$.mp. OR exp seed/ OR cereal$.mp. OR exp cereal/ OR grain$.mp. OR exp grain/ OR fermented.mp. OR dairy.mp. OR ‘non dairy’.mp. OR prune$.mp. OR kiwi$.mp. OR kefir.mp OR tea.mp. OR coffee.mp.   **NOT**  Isolate$.ti,ab. OR extract$.ti,ab.  **AND**  exp randomized controlled trial/ OR exp controlled clinical trial/ OR random$.ti,ab. OR exp randomization/ OR exp intermethod comparison/ OR placebo.ti,ab. OR (compare or compared or comparison).ti. OR ((evaluated or evaluate or evaluating or assessed or assess) and (compare or compared or comparing or comparison)).ab. OR (open adj label).ti,ab. OR ((double or single or doubly or singly) adj (blind or blinded or blindly)).ti,ab. OR exp double blind procedure/ OR parallel group$1.ti,ab.  OR  (crossover or cross over).ti,ab. OR ((assign$ or match or matched or allocation) adj5 (alternate or group$1 or intervention$1 or patient$1 or subject$1 or participant$1)).ti,ab. OR (assigned or allocated).ti,ab. OR (controlled adj7 (study or design or trial)).ti,ab. OR (volunteer or volunteers).ti,ab. OR exp human experiment/ OR trial.ti. OR exp prospective study/ OR 'single arm'.ti,ab. OR 'intervention study'.ti,ab.   exp comparative study/   **NOT**  (random$ adj sampl$ adj7 ('cross section$' or questionnaire$1 or survey$ or database$1)).ti,ab. not (comparative study/ or controlled study/ or randomi?ed controlled.ti,ab. or randomly assigned.ti,ab.) OR Cross-sectional study/ not (randomized controlled trial/ or controlled clinical study/ or controlled study/ or randomi?ed controlled.ti,ab. or control group$1.ti,ab.) OR (((case adj control$) and random$) not randomi?ed controlled).ti,ab.   OR (Systematic review not (trial or study)).ti. . OR 'Random field$'.ti,ab. OR (random cluster adj3 sampl$).ti,ab. OR (review.ab. and review.pt.) not trial.ti. OR 'we searched'.ab. and (review.ti. or review.pt.) OR 'update review'.ab. OR (databases adj4 searched).ab. OR  (rat or rats or mouse or mice or swine or porcine or murine or sheep or lambs or pigs or piglets or rabbit or rabbits or cat or cats or dog or dogs or cattle or bovine or monkey or monkeys or trout or marmoset$1).ti. and animal experiment/ OR Animal experiment/ not (human experiment/ or human/)  *8726 records* | Title, abstract, keywords:  Food OR drink OR herb OR spice  **AND**  Search all text:  Constipation  *1142 records* | - |

**Table S2: GRADE critical outcomes**

- Response to treatment
- Stool frequency
- Stool consistency
- Global symptoms
- Straining
- Hard stools
- Incomplete evacuation
- Sensation of anorectal obstruction/blockage
- Manual maneuvers
- Adverse events: abdominal pain/discomfort
- Adverse events: bloating
- Adverse events: flatulence
- Quality of life
